# Tumor microenvironment targeted nano-drug delivery systems for multidrug resistant tumor therapy

**DOI:** 10.7150/thno.103636

**Published:** 2025-01-02

**Authors:** Xinyue Shao, Xiaoling Zhao, Binghao Wang, Jiahui Fan, Jinping Wang, Hailong An

**Affiliations:** Key Laboratory of Molecular Biophysics of Hebei Province, Institute of Biophysics, School of Health Sciences and Biomedical Engineering, Hebei University of Technology, 300401, Tianjin, PR China.

**Keywords:** Tumor microenvironment, Nano-drug delivery systems, Multidrug resistance, Cancer therapy

## Abstract

In recent years, nano-drug delivery systems (Nano-DDS) that target the tumor microenvironment (TME) to overcome multidrug resistance (MDR) have become a research hotspot in the field of cancer therapy. By precisely targeting the TME and regulating its unique pathological features, such as hypoxia, weakly acidic pH, and abnormally expressed proteins, etc., these Nano-DDS enable effective delivery of therapeutic agents and reversal of MDR. This scientific research community is increasing its investment in the development of diversified systems and exploring their anti-drug resistance potential. Therefore, it is particularly important to conduct a comprehensive review of the research progress of TME-targeted Nano-DDS in recent years. After a brief introduction of TME and tumor MDR, the design principle and structure of liposomes, polymer micelles and inorganic nanocarriers are focused on, and their characteristics as TME-targeted nanocarriers are described. It also demonstrates how these systems break through the cancer MDR treatment through various targeting mechanisms, discusses their synthetic innovation, research results and resistance overcoming mechanisms. The review was concluded with deliberations on the key challenges and future outlooks of targeting TME Nano-DDS in cancer therapy.

## 1. Introduction

In the relentless fight against cancer, the development of targeted treatments has been crucial for progress. However, one of the major challenges facing modern oncology is the phenomenon of multidrug resistance (MDR), which greatly limits the effectiveness of traditional chemotherapy drugs and targeted drugs 1. MDR arises through various mechanisms, such as altered drug targets, enhanced drug efflux, and constraints within the tumor microenvironment (TME) 2. The TME is a dynamic and intricate ecosystem, comprising diverse cell types (e.g., tumor cells, immune cells, fibroblasts) and non-cellular elements (e.g., extracellular matrix, growth factors, cytokines), which fosters tumor cell evasion of treatment and drug resistance 3, 4. Conditions like hypoxia, acidosis, dense ECM, abnormal vascularity, and an immunosuppressive environment contribute to a hostile yet conducive setting for tumor cell survival and the progression of drug resistance 5, 6.

To surmount the challenge of MDR, researchers are increasingly looking to nano-drug delivery systems (Nano-DDS) as a promising strategy to overcome MDR. Nano-DDS often offers several advantages over traditional drug delivery methods, including enhanced penetration and retention (EPR) effects, targeted ability to deliver drugs to specific tumor sites, as well as the capability to co-deliver multiple drugs or therapeutics in a controlled and sustained manner 7, 8. By targeting the unique characteristics of TME, Nano-DDS can be designed to penetrate dense ECMs, bypass efflux pumps, and target specific compartments within tumor cells or specific cell types within TME 9. Furthermore, by integrating stimuli-responsive materials, Nano-DDS can be designed to release its therapeutic payload in response to local environmental signals, including variations in pH, hypoxia, or the presence of particular enzymes, thereby augmenting drug efficacy and mitigating systemic toxicity 10-12.

**Figure 1:**
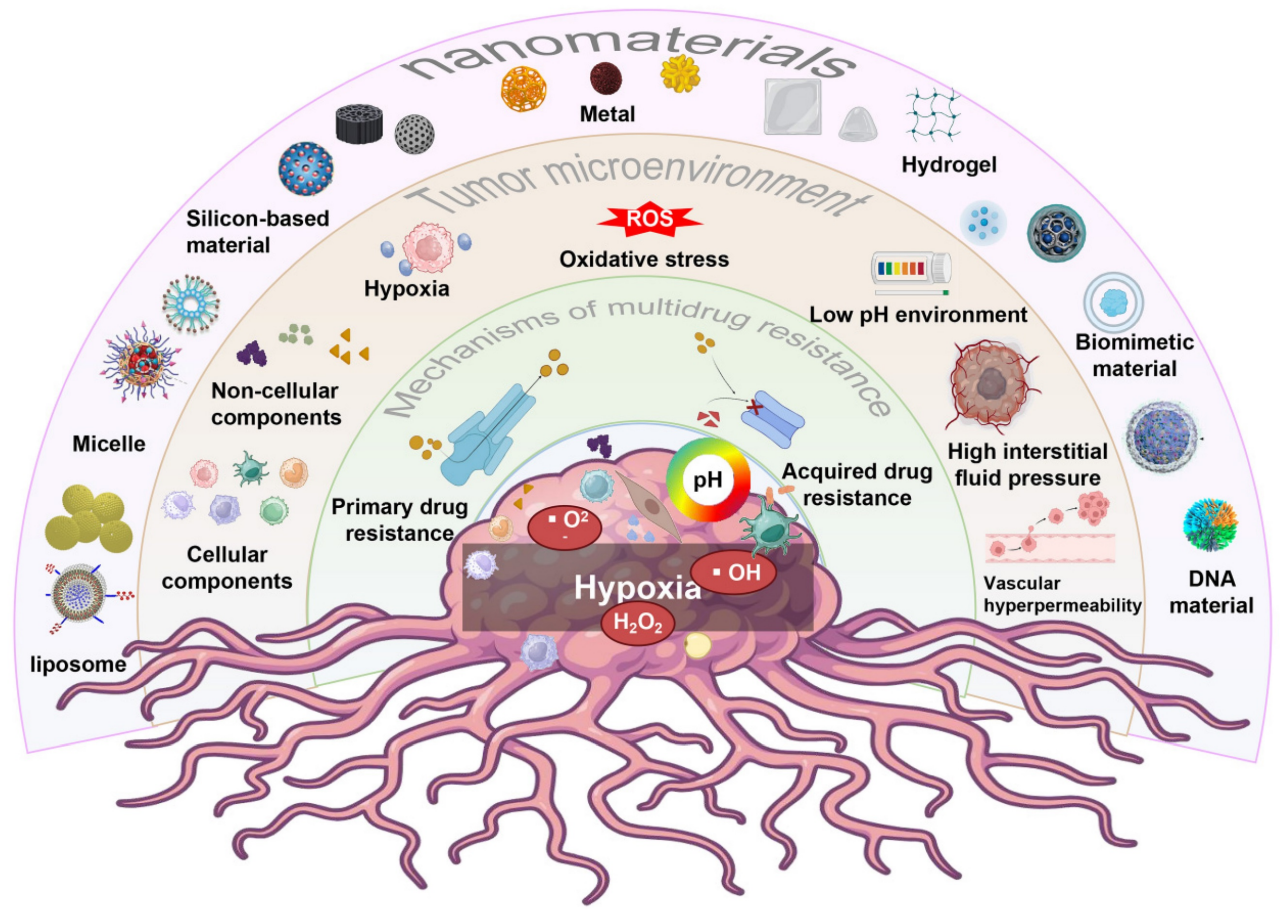
Schematic diagram of multiple Nano-DDS used to treat MDR tumors by targeting TME, including the mechanism of MDR, the composition of TME, and multiple types of nanoparticles.

In this review, we will discuss recent advances in Nano-DDS that overcome MDR by targeting the TME. We will explore the characteristics of TME, the various mechanisms of tumor MDR in TME, and how Nano-DDS can be customized to overcome these barriers. In addition, we will review recent advances in the field of Nano-DDS, including their EPR effects, targeted specific cell types in TME, and the targeted delivery of drugs to specific cell types within the TME, and stimulus-responsive release mechanisms. Finally, we will discuss the challenges and future directions in the tumor treatment field, with a focus on translating Nano-DDS from the laboratory to clinical applications to improve the quality of life of cancer patients (**Figure 1**).

## TME and MDR

### Overview of TME

The malignant transformation of tumor is a complex multi-stage process, which is rooted in the instability of the genome, resulting in the cell differentiation obstruction, and then transformed into aggressive cancer cells. Despite advances in treatment, distant metastasis is still the leading cause of death from cancer. Stephen Paget's "seed and soil" theory reveals the mechanism of metastasis: potential cancer cells (seeds) can select and adapt to a specific tissue environment (soil) to grow and spread, emphasizing the critical role of the TME 13. TME, as an important stage for tumor growth and metastasis, is composed of various cell types, soluble factors, and ECM. In this complex environment, tumor cells interact closely with surrounding stromal cells, immune cells, and the vascular system by releasing and responding to various signaling molecules 14, 15. MDR poses a major challenge in cancer therapy, leading to treatment failure and disease progression 16. The TME contributes to MDR by fostering an environment that enhances tumor cell survival and resistance. By providing a protective niche, the TME supports cancer cell subpopulations with stem-like properties, making them less susceptible to chemotherapy 17-20. The TME plays an important role in the tumor MDR through its complex interactions between cellular and non-cellular components. Therefore, a thorough understanding of the relationship between TME and MDR is of great significance for developing new anti-tumor treatment strategies and improving treatment effects.

#### Composition of TME

**Figure 2:**
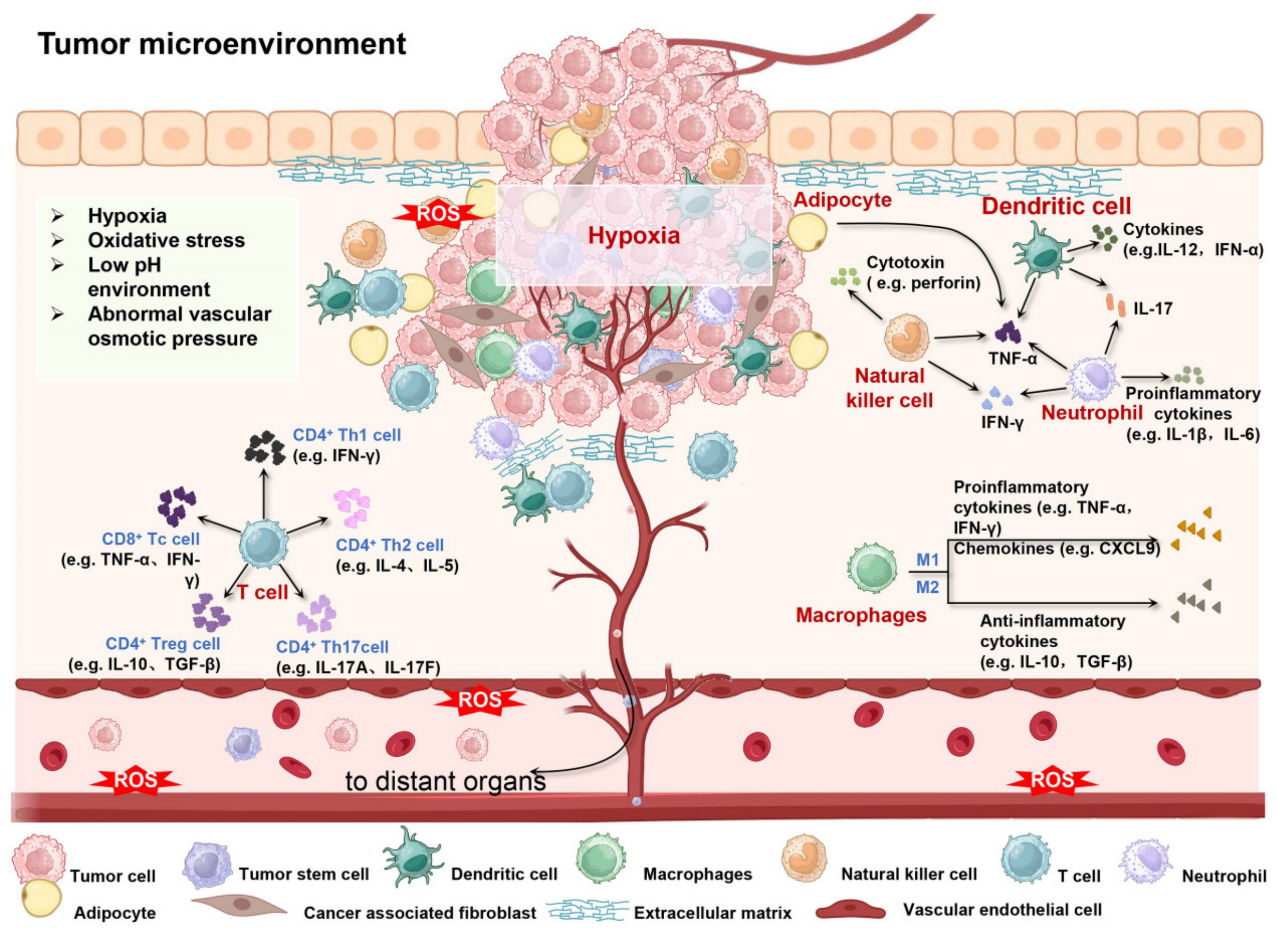
Schematic diagram of the interaction between cellular/non-cellular components and physical/chemical environment in TME. Created using BioRender software (http://biorender.com).

The TME constitutes a highly dynamic and intricate ecosystem, encompassing a diversity of cellular and acellular components. It is composed of tumor cells, stromal cells (which include fibroblasts, various immune cells such as T cells, B cells, macrophages, dendritic cells, etc., and endothelial cells), the Extracellular Matrix (ECM) comprising collagen, proteoglycans, hyaluronic acid, etc., and the vascular system, which involves tumor angiogenesis, vascular normalization, and abnormal vessels. Additionally, it features an immunosuppressive and immune-escape-prone immune microenvironment 4, 21. This multicellular and multi-layered ecosystem is not only rich but also complex, providing a breeding ground for tumor cells to evade therapeutic interventions and promote the development of MDR 22, 23. The components of TME are interwoven to form complex communication networks with each other and with the heterogeneous cancer cells themselves, which together promote tumor growth, invasion and metastasis, and increase the microenvironment of drug resistance (**Figure 2**).

##### (1) Cell components

The cell composition of the TME is very rich and complex, mainly including tumor cells themselves and various cell types around them, which play a crucial role in the occurrence, development and metastasis of tumors. Through complex interactions and regulation, these cell components together constitute a dynamic ecosystem that has a profound impact on tumor growth, invasion and therapeutic effectiveness.

Tumor cells. Tumor cells are a core component of the TME that forms tumor tissue through uncontrolled proliferation and escape from apoptosis 24, 25. These cells orchestrate the supportive tumor environment by enlisting and reprogramming non-cancerous host cells, restructuring the vascular network and ECM, and collaboratively fueling tumor progression and metastasis 16, 26.

Immune cells. The immune cell system is large and versatile, mainly divided into two categories: adaptive immune cells (AICs) (such as CD8^+^ T cells, CD4^+^ T cells, B cells, etc.) and myeloid immune cells (MICs) (such as macrophages, neutrophils, monocytes, dendritic cells, mast cells, eosinophils, myeloid derived suppressor cells (MDSC), etc.) 27-32. AICs can directly kill or inhibit tumor cells by recognizing antigens on their surface and initiating adaptive immune responses 33. However, despite AICs' ability to recognize and eliminate pathogens and non-self-antigen-expressing cells, many cancers can prevent or neutralize immune attacks early in tumor development 34. For instance, studies in melanoma patients have found that early in tumor progression, despite the infiltration of T cells, these T cells are often in a state of functional exhaustion, manifested by reduced proliferative capacity, decreased cytokine production, and impaired killing activity, which may be due to the combination of immunosuppressive factors released by melanoma cells or other factors in the TME 35. MICs also have complex functions in the TME. On the one hand, myeloid immune cells, as an essential defense line of the immune system, can exert an anti-tumor effect by directly phagocytosing and killing tumor cells or inhibiting their proliferation 36. On the other hand, MICs may also be "reshaped" by the TME and turn into factors that can promote tumor growth and metastasis. During this transition, MICs may secrete a series of immunosuppressive factors, such as IL-10, ROS, iNOS, arginase 1, and TGF-β, which inhibit the activity of T and NK cells, thereby weakening the anti-tumor ability of the immune system 37. At the same time, they may also express immune checkpoint molecules such as PD-L1, further hindering immune cell activation and function 38. In addition, MICs produce inflammatory mediators such as IL-1β, TNF-α, and IL-6, which may initially help initiate an anti-tumor immune response. However, when these mediators persist within the TME, they tend to exacerbate the inflammatory response, fostering tumor angiogenesis, immune evasion, and ultimately facilitating tumor growth and dissemination 39. For example, macrophage subpopulations in early lung cancer tissue show specific molecular signatures, including high PPARγ expression, reduced CD86 expression, and increased PD-L1 levels. 40.

Tumor stromal cells. Tumor stromal cells including cancer-associated fibroblasts (CAFs), adipocyte, *etc.* 41. CAFs are one of the most abundant stromal cells in the TME, and their high activity significantly promotes the proliferation, invasion and metastasis of tumor cells through the release of various growth factors (such as TGF-β), cytokines (such as IL-6, IL-8) and chemokines 42. Some tumors, such as hepatocellular carcinoma, are the result of abnormal activation of fibroblasts, especially in fibrotic or cirrhotic liver 43. CAFs can also facilitate the migration of tumor cells by reshaping ECM, for example, fibrosis in TME leads to tissue stiffness, which is significantly associated with poor survival in patients with pancreatic and breast cancer 44, 45. Moreover, adipocytes show a dynamic and reciprocal relationship with tumor cells in the TME. By secreting adipokines, they can regulate the metabolism and growth of tumor cells. Additionally, by modifying the ECM, adipocytes can also influence the migration and invasion of tumor cells 46-47. Cancer-associated adipocytes (CAAs) include the following broad categories, such as intratumoral adipocytes, peritumoral adipocytes, recruitment adipocytes, and mesenchymal stem cells (MSCs) that differentiate into adipocytes or adipose-like cells that store large amounts of energy-rich lipids. Studies have shown that CAAs directly or indirectly affects TME through paracrine, hormone and pro-inflammatory cytokines (such as CCL2, CCL5, etc.), aggravating cancer invasion and immune evasion, and displaying dysregulated pro-inflammatory properties 48. For example, CAAs can induce a fibroblast-like transformation in breast cancer cells and increase immunosuppressive CAFs 49.

Vascular cells. Vascular cells including vascular endothelial cells (ECs) and lymphatic endothelial cells (LECs). Vascular endothelial cells (ECs), as the basic building block of the vascular system 50, are derived from two main ways. Firstly, they are differentiated from endothelial progenitor cells (EPCs) in bone marrow or other tissues, which have high proliferation and differentiation potential and can be recruited to the tumor site and participate in the formation of new blood vessels under specific conditions 51; The second is to respond to the needs of tumor growth through the proliferation and remodeling of the existing blood vessel network, which is the process of angiogenesis 52. These ECs are intricately arranged to form a complex network of blood vessels, providing the essential blood supply to rapidly proliferating tumor cells. This ensures that tumor cells receive adequate oxygen, nutrients, and growth factors, thereby supporting their sustained growth and expansion. In this process, ECs are not only passive vascular wall components, they also actively participate in the regulation of TME by secreting a variety of growth factors (such as VEGF, FGF, etc.) and cytokines (such as IL-6, IL-8, etc.) 53. These factors can not only promote the proliferation and survival of tumor cells, but also induce the recruitment and activation of other stromal cells, such as fibroblasts and immune cells, thereby further exacerbating the immunosuppression and inflammatory response within the TME, and ultimately creating favorable conditions for tumor growth and metastasis 54. LECs, on the other hand, regulate lymph fluid flow within tumor tissue, maintaining tissue fluid balance and immune cell migration 55. LECs maintains tumor homeostasis by forming a network of lymphatic vessels that drain excess fluid, waste, and immune cells from tumor tissue to the peripheral lymphatic system. However, when they are dysfunctional by the TME, lymph fluid retention exacerbates tumor edema, inflammation, and promotes the spread of tumor cells through the lymphatic system 56-57. For example, in breast cancer studies, ECs and LECs proliferate abnormally in tumor tissues, and ECs secretes high levels of VEGF to promote angiogenesis and support cancer cell proliferation. LECs dysfunction obstructs lymphatic return and promotes cancer cell metastasis through the lymphatic system to distant tissues such as axillary lymph nodes 58.

Cancer stem cells (CSCs). The roots of most cancers can be traced to CSCs that carry characteristic surface markers similar to normal stem cells (NCSs), such as CD44, CD90, and CD133. These CSCs act as core builders of the TME, self-renewing pathophysiological processes that drive tumor development with the support of multiple non-cancer cells 59. CSCs show a high degree of plasticity and immunomodulatory ability, which can skillfully evade the surveillance of the immune system and become the main culprit of primary cancer and immunotherapy resistance 60. CSCs achieve immune regulation through multiple mechanisms, including bidirectional cytokine release, extracellular vesicle-mediated intercellular communication, and fusion with stromal cells, which together promote CSC's immune escape strategy 61, 62. It is particularly striking that CSCs can enter a dormant state at the same time as immune escape, further increasing its viability 63. CSCs can also fuse with a variety of microenvironmental cells (such as fibroblasts, macrophages, MDSCs, and MSCs) to generate abnormal cells with stem-like properties that are closely related to tumor initiation, progression, and metastasis potential 64, 65. CSCs play a key role in promoting tumor metastasis and heterogeneity, which is also an important reason for their resistance to chemotherapy, radiotherapy and immunotherapy.

##### (2) Non-cellular components

The non-cellular components of the TME also play an important role in the process of tumor initiation, development and metastasis. These non-cellular components mainly include ECM and its growth factors (GFs), cytokines, chemokines and so on, which perform the function of information exchange.

ECM. ECM, composed of collagen, glycoprotein, etc., creates a stable environment for tumor cells, maintains tissue integrity, and regulates its growth, invasion and metastasis 66. During the complex process of tumor progression, the composition of the ECM changes significantly, which is manifested by decreased expression of attachment proteins such as LAMB1 and LAMC1, while at the same time, the expression of migration-related proteins such as FN1 and COMP increases significantly 67. This dynamic change not only profoundly affects the migration ability of tumor cells, but also significantly regulates the role of ECM in chemotherapy resistance and tumor proliferation. It is worth noting that the specific components of ECM are closely related to chemotherapy drug sensitivity. For example, in triple-negative breast cancer (TNBC), the hardness of ECM becomes an important factor affecting the efficacy of doxorubicin treatment 68. Therefore, ECM and its relationship with tumor drug resistance have become a promising direction in the future research of tumor therapy.

Growth factors (GFs). As an important protein or polypeptide secreted by cells, GFs have a wide range of regulatory effects in organisms, especially in the occurrence and development of tumors, they play an indispensable role 14. These GFs promote cell growth, proliferation and differentiation through complex signal networks, which have a profound impact on the growth rate, malignancy and invasion ability of tumors 69. For example, vascular endothelial growth factors (VEGFs) released by neutrophils are a strong catalyst for angiogenesis, promoting the construction of neovascular networks within tumors, ensuring adequate oxygen and nutrients for tumors, and accelerating tumor growth and spread 53, 70. At the same time, GFs secreted by mast cells are also actively involved in regulation, that is, decomposing ECM through matrix metalloproteinases (MMPs) (such as MMP2 and MMP9), releasing VEGF, bFGF, and other angiogenic factors to build a more abundant tumor blood supply network 71. In addition, the role of GFs goes far beyond promoting angiogenesis, for example, transforming growth factor-β (TGF-β) 72, platelet-derived growth factor (PDGF) 73, fibroblast growth factor (FGF) 74, and other members of the GF family can also activate stromal cells such as fibroblasts, triggering structural remodeling and functional adjustment of tumor stroma. These changes not only optimized the growth environment of tumor cells, but also profoundly shaped the malignant phenotype and biological characteristics of tumors by affecting the differentiation trajectory of tumor cells, demonstrating the multifaceted and critical role of GF in tumor regulation.

Cytokines. Cytokines are a class of small molecular proteins with a wide range of biological activities synthesized and secreted by immune cells and some non-immune cells (endothelial cells, epidermal cells, fibroblasts, etc.) after stimulation, including the interleukin (IL) family (IL-1β, IL-2, IL-6, IL-6, IL-6, and so on). IL-7, IL-8, IL-10, IL-12), tumor necrosis factor (TNF) family (TNF-α, TNF-β), interferon (IFN) family (IFN-α, IFN-β, IFN-γ), etc 75-77. Cytokines play a crucial role in the TME, which regulate tumor growth, invasion and metastasis through multiple pathways. For example, IL-1β, as a pro-inflammatory cytokine, is up-regulated in tumors such as breast cancer, promoting tumor growth and invasiveness, and is associated with poor prognosis 78. IL-2, as a T cell growth factor, enhances T cell activity to inhibit tumor growth, and its clinical application has shown therapeutic effects on specific types of tumors 79. On the other hand, IL-6 promotes tumor growth, angiogenesis and immune escape by regulating immune balance and activating multiple signaling pathways, and becomes a potential target for anti-tumor therapy 80. In addition, TGF-β, as a multifunctional cytokine, promotes tumor cell survival, proliferation and immune escape in TME, which is still a research hotspot despite the challenges of its targeted therapy 81. The complex mechanism of action of these cytokines in TME not only reveals the deep-seated law of tumor occurrence and development, but also provides an important basis for developing new anti-tumor strategies.

Chemokines. Chemokines are a class of small molecular proteins or peptides that attract and activate white blood cells. These chemokines are divided into four main subfamilies based on their structural characteristics: C chemokines subfamily (XCL1 and XCL2), CC chemokines subfamily (CCL2 (MCP-1), CCL3, CCL4, CCL5 (RANTES)), CXC chemokines subfamily (CXCL8 (IL-8), CXCL12 (SDF-1), CXCL9, CXCL10 CXCL11) and CX3C chemokine subfamily (CX3CL1) 82. By binding to their receptors, chemokines form a complex network to regulate cell migration and distribution, thus playing an important role in physiological and pathological processes 83. For example, CCL2 (MCP-1) in the CC chemokine subfamily is a key chemokine for monocytes and macrophages, and is involved in inflammatory response and recruitment of immune cells in the TME 84. However, CXCL8 (IL-8) in the CXC chemokine subfamily mainly chemotactic neutrophils and participate in the activation process of other immune cells 85. In addition, CXCL12 (SDF-1), as a ligand for the CXCR4 receptor, plays an important role in the migration and homing of a variety of cells 86. Chemokines regulate cell migration and distribution through the formation of complex network relationships, which not only play a role in physiological processes such as cell growth, development, differentiation and apoptosis, but also play a key role in pathological processes such as inflammation, pathogen infection, wound repair and tumor formation and metastasis. Therefore, the in-depth study of the function of chemokines and their receptors is of great significance for understanding the mechanism of cell migration and developing new therapeutic methods and diagnostic tools.

#### Physical and chemical environment of TME

The physical and chemical environment of the TME refers to the specific physical and chemical conditions in which tumor cells and their surrounding stromal cells are located, which have important effects on tumor growth, invasion, metastasis and therapeutic response. The following is a detailed analysis of the physical and chemical environment of the TME:

(1) Hypoxic environment. TME is known for its unique hypoxic properties, which are due to the delayed development of the vascular network that accompanies the rapid proliferation of tumor tissue, as well as the distortion of the vascular structure within the tumor and the blockage of blood flow 87. In order to maintain survival and expansion, tumor cells significantly upregulate the expression of hypoxia-inducing factors (HIF), specifically HIF-1α and HIF-2α, which regulate the angiogenesis process in a complex manner. Specifically, HIF-1, a key bHLH transcription factor, is activated in response to hypoxia, triggering the expression of a series of genes closely associated with angiogenesis and cell survival in response to hypoxia challenges 88. As an intracellular “oxygen regulator”, HIF-1 not only promotes the proliferation of tumor cells, but also shapes drug-resistant phenotypes by regulating biochemical metabolic pathways, and enhances chemotherapy resistance 89. In addition, the activation of carcinogenic signaling pathways common in solid tumors, such as Ras and PI3K/AKT, and the inactivation of tumor suppressors, such as LKB1, PTEN, and TSC2/1, further exacerbate drug resistance by activating HIF-1 90, 91. Given its central role in regulating multiple metabolic pathways such as amino acid metabolism, lipid metabolism, gluconeogenesis, and the tricarboxylic acid cycle, HIF-1α is a key factor in maintaining cancer cell survival and resistance to therapy. Therefore, prior to the design and implementation of effective tumor therapy, it is of great significance to deeply explore the pathological mechanism of HIF-1α expression and develop targeted therapies for it to improve the therapeutic effect and overcome drug resistance.

(2) Low pH environment. The TME tends to be acidic (pH ~6.2 to 6.5), with pH significantly lower than normal tissue levels (pH ~7.4) 92, 93. The core of this phenomenon is that tumor cells are abnormally active in metabolism, producing a large number of acidic metabolic byproducts such as lactic acid (10-30 mM in TME vs 1.5-3.0 mM in normal physiological environment), while the lymphatic system within the tumor is blocked, resulting in these acidic substances cannot be effectively discharged from the body, and thus accumulate locally 94. This acidic environment not only directly activated oncogenes, but also prompted adaptive changes in cell metabolism in response to adverse conditions 95. In addition, the acidic microenvironment further regulates immune surveillance and tumor progression by inducing tumor-associated macrophages (TAMs) to become M2-type polarized and weakening the cytotoxicity of infiltrating T cells 96. From a therapeutic perspective, the pH gradient inside and outside the tumor cells forms a physical barrier that is impenetrable due to the altered ionization state of weakly basic chemotherapy drugs (vincristine, doxorubicin and paclitaxel) in the acidic extracellular environment, which significantly reduces the uptake rate of drugs and poses a therapeutic challenge 97.

(3) Oxidative stress. Oxidative stress in the TME is a complex biological phenomenon, which refers to the imbalance between excessive reactive oxygen species (ROS) produced by tumor cells and their surrounding stromal cells during metabolism and the antioxidant defense system 98. This imbalance leads to the accumulation of ROS in cells, which leads to a series of oxidative damage and signal transduction changes, which can promote a variety of adaptive changes in tumor cells, including increased resistance to chemotherapy drugs 99. High levels of ROS can induce DNA damage, gene mutation and epigenetic changes in tumor cells, which may directly lead to changes in drug targets or reconfiguration of drug metabolism pathways, so that tumor cells can escape the attack of chemotherapy drugs 100. In addition, oxidative stress can also activate a series of signal transduction pathways related to drug resistance, such as MAPK, PI3K/Akt and NF-κB, etc. And the activation of these pathways will further up-regulate the expression of drug-resistance related genes and promote the development of drug resistance in tumor cells 101. Therefore, ROS species in the regulated TME is an important means to solve the drug resistance of tumor cells.

(4) Abnormal vascular osmotic pressure. Abnormal vascular osmotic pressure refers to increased vascular permeability and interstitial fluid pressure (IFP) within the TME, both of which significantly affect tumor growth, invasion, and treatment response. Tumors often exhibit high vascular permeability due to factors such as distorted vascular structures, incomplete endothelial cell layers, and basement membrane rupture 102. For example, in breast cancer, tumor blood vessels are disorganized, with enlarged endothelial cell spaces, allowing key molecules like plasma proteins and GFs to easily cross the blood vessel wall and enter the tumor stroma 103. This leakage creates a hypertonic microenvironment rich in nutrients and growth factors, promoting rapid tumor cell proliferation and aggressive growth, while also exacerbating the TME's deterioration. Worse yet, tumor cells can exploit this high permeability to enter the systemic circulation, spreading to other parts of the body and significantly increasing the risk of distant metastasis, thus posing a serious threat to patient health 103. Additionally, the vascular network within tumors is often dysfunctional, with unbalanced blood vessel formation, enlarged capillary spacing, arteriovenous shunting, and obstructed lymphatic drainage, all of which contribute to high IFP 104. These factors not only create the unique TME but also hinder the effectiveness of conventional therapies such as chemotherapy, targeted therapy, and radiotherapy, while promoting drug resistance 105. Specifically, high IFP acts as a physical barrier, impeding the penetration of therapeutic drug molecules and immune cells into tumor tissues, thus limiting their distribution and effectiveness and directly weakening therapeutic outcomes. This presents a significant challenge for cancer treatment 106. Therefore, it is crucial to consider the impact of these factors in cancer treatment and implement strategies to reduce vascular permeability and interstitial fluid pressure to enhance therapeutic efficacy and reduce the risk of metastasis

### Overview of MDR

MDR refers to the phenomenon that tumor cells develop resistance to one anti-tumor drug, but also cross-resistance to anti-tumor drugs with completely different structures and mechanisms of action. MDR is an important problem in the treatment of cancer, which seriously affects the therapeutic effect and the survival rate of patients. There are two main types of MDR: 1) Primary drug resistance: tumor cells develop resistance to anti-tumor drugs at the initial stage of treatment. In this case, the tumor cells are already resistant to the drug before they are exposed to it. For instance, genes encoding ABC transporter proteins, such as P-glycoprotein (P-gp), are overexpressed, leading to the active efflux of drugs from the cells, thereby reducing intracellular drug accumulation. In the case of breast cancer, high expression of P-gp contributes to primary resistance to multiple chemotherapeutic drugs in some patients 107. 2) Acquired drug resistance: during the course of chemotherapy, tumor cells that were originally sensitive to drugs gradually become resistant to drugs. This condition is usually caused by genetic alterations or epigenetic changes in tumor cells under the pressure of the drug, thereby acquiring resistance to the drug 108. For example, in the treatment of lung cancer, epidermal growth factor receptor (EGFR) tyrosine kinase inhibitors (TKI) such as gefitinib are effective initially, but after long-term use, tumor cells may evade the inhibition of the drug through T790M mutations in the EGFR gene, leading to the development of drug resistance 109. In addition, tumor cells may enhance resistance to DNA-damaging drugs by up-regulating the expression of DNA repair enzymes (such as Topoisomerase Iα), or by activating alternative survival signaling pathways (such as the PI3K/AKT/mTOR pathway) to bypass drug targets 110.

### Mechanism of TME mediating the development of MDR in tumors

As a complex and dynamic ecosystem, the TME play a crucial role in mediating the development of drug resistance in cancer cells, especially in promoting the formation of MDR. MDR is a major challenge in cancer treatment, which limits the effectiveness of various treatments such as chemotherapy and targeted therapies. The following are the main mechanisms by which TME mediates the development of MDR and their elaboration.

(1) ECM remodeling and drug penetration. The ECM provides structural and biochemical support to cells within the TME. In tumors, ECM remodeling is significant, characterized by increased deposition of ECM components (e.g., collagen, fibronectin), cross-linking, and stiffening. This remodeling, mediated by cancer cells and stromal cells like CAFs, is facilitated by enzymes such as lysyl oxidase (LOX) and transglutaminases 111. The denser, more complex ECM creates a physical barrier to drug delivery. It hinders the diffusion and convection of chemotherapeutic agents from blood vessels to cancer cells, leading to inadequate drug concentrations at the tumor site. Additionally, high IFP resulting from ECM remodeling further impedes drug transport by collapsing blood vessels and reducing transvascular transport. Interactions between ECM components and cancer cells via integrins and other receptors activate signaling pathways that promote survival, proliferation, and MDR. These include FAK, Src, and MAPK signaling cascades, which enhance the expression of drug resistance genes and anti-apoptotic proteins 112.

(2) Immune cells and MDR. The TME is infiltrated by various immune cells, including TAMs, Tregs, and MDSCs, which contribute to MDR. TAMs, especially those polarized to the M2 phenotype, secrete anti-inflammatory cytokines (e.g., IL-10, TGF-β) and growth factors that promote tumor growth, angiogenesis, and survival. These immune cells enhance drug resistance by releasing cytokines and chemokines that activate survival pathways in cancer cells, such as NF-κB and STAT3 signaling 113. They also induce the expression of drug efflux pumps and anti-apoptotic proteins in cancer cells. The cell-to-cell interaction between immune cells and cancer cells facilitates the exchange of signaling molecules that further promote MDR 114.

(3) Role of CAFs in MDR. CAFs are a key component of the tumor stroma and play a crucial role in tumor progression and drug resistance. CAFs secrete soluble factors, including growth factors (e.g., TGF-β, HGF), cytokines, and chemokines, which activate pro-survival signaling pathways in cancer cells, such as PI3K/Akt and MAPK pathways, enhancing their resistance to chemotherapy 115. Additionally, CAFs remodel the ECM by producing and reorganizing components like collagen, fibronectin, and hyaluronan, increasing ECM density and stiffness. This creates a physical barrier that hinders the penetration and diffusion of chemotherapeutic agents. CAFs also secrete MMPs, which modify the ECM and release bioactive molecules that promote tumor survival and MDR 116.

(4) Hypoxia-induced MDR. Under hypoxic conditions, hypoxia-inducible factors (particularly HIF-1α) upregulate the expression of drug efflux pumps such as P-glycoprotein and multidrug resistance-associated proteins, which pump chemotherapeutic agents out of cancer cells, reducing intracellular drug accumulation and efficacy, thereby contributing to MDR 117. Hypoxia also induces genetic instability and selects for more aggressive cancer cell phenotypes that are resistant to apoptosis 118. This environment enhances DNA repair mechanisms in cancer cells, allowing them to survive chemotherapy-induced DNA damage. Additionally, hypoxia stimulates the production of VEGF, promoting angiogenesis and further modifying the TME to support tumor survival and MDR 119.

(5) Acidic pH and drug resistance. The acidic TME affects drug uptake and activity in several ways. First, the protonation of weakly basic chemotherapeutic drugs in acidic conditions reduces their ability to penetrate cell membranes, a phenomenon known as “ion trapping”. This leads to decreased intracellular drug concentrations. Second, acidic pH alters the stability and efficacy of certain drugs, reducing their cytotoxic effects. Furthermore, acidity influences the activity of enzymes involved in drug metabolism and the expression of drug transporters, further promoting MDR. The acidic environment also induces adaptive responses in cancer cells, such as activation of acid-sensing ion channels and stress response pathways, which enhance cell survival under chemotherapy-induced stress 120.

In conclusion, there is a close relationship between TME and MDR, and the two influence and promote each other. A thorough understanding of this relationship and the exploration of effective coping strategies are of great significance for improving the efficacy of tumor treatment and patient prognosis.

## Nano-DDS with targeted TME to overcome tumor MDR

The research of Nano-DDS targeting TME to overcome tumor MDR is an important direction in the field of cancer therapy 121. As a novel drug delivery system, Nano-DDS has several advantages that help it target TME and overcome MDR. 1) Targeting: Nano-DDS can be designed to carry specific ligands or antibodies on its surface, so as to achieve specific recognition and binding to tumor cells and improve the targeting of drugs 122. 2) Slow and controlled release: Nano-DDS can control the release rate of drugs, achieve slow and controlled release of drugs, reduce frequent drug administration and side effects, and maintain the effective concentration of drugs at the tumor site 123. 3) Penetrability: By adjusting the size of the nanoparticles, Nano-DDS can optimize their match with the TME barrier and thus more efficiently cross the physical barriers in the TME. At the same time, adjusting its shape to a more streamlined or specifically targeted form not only reduces the friction resistance with the surrounding environment, but also enhances the affinity with the target cells, further promoting the penetration efficiency. In addition, by modifying the surface to carry specific ligands or charges, Nano-DDS can actively identify and bind to receptors or specific molecules on tumor cells for precise targeting and efficient penetration 124.

With the deepening of the research on Nano-DDS, its application in cancer therapy is becoming increasingly promising (Table 1). In the future, Nano-DDS are expected to become one of the important means to overcome tumor MDR and provide more effective and safe treatment for cancer patients. At the same time, with the continuous development of nanotechnology and materials science, the performance and stability of Nano-DDS will continue to improve, providing a strong guarantee for its wide clinical application.

### Lipid-based Nano-DDS

Liposomes, as a long-established and promising Nano-DDS, have extraordinary application value due to their unique structure. The structure is usually a spherical structure with a hydrophilic material as the core, surrounded by a double layer membrane, which is mainly composed of phospholipids and other amphiphilic lipid materials 125. The size of liposomes is flexible and can be precisely controlled in a wide range from 20 nm to 1000 nm. According to the number and particle size of bilayer membranes, liposomes are divided into two categories. The first one is the monolayer liposome, which encompasses three subtypes: small monolayer (with a diameter of 20-100 nm), large monolayer (with a diameter ranging from 100 to 1000 nm), and giant monolayer (with a diameter exceeding 1000 nm), designed to meet the packaging requirements of diverse drugs. The second one is multilayered liposome, with complex onion-like structure, especially those with a diameter of more than 500 nanometers, provides ideal carriers for drugs that require larger volumes or special slow-release effects 126. In the face of complex tumor microenvironment, liposomes show remarkable adaptability and potential. By reasonably designing the surface properties, size, charge, and the targeted molecules carried by liposomes, their penetration, retention and uptake by specific cells in tumor tissues can be precisely regulated 127, so as to achieve accurate drug delivery and increase the retention time of drugs in cells.

**Figure 3:**
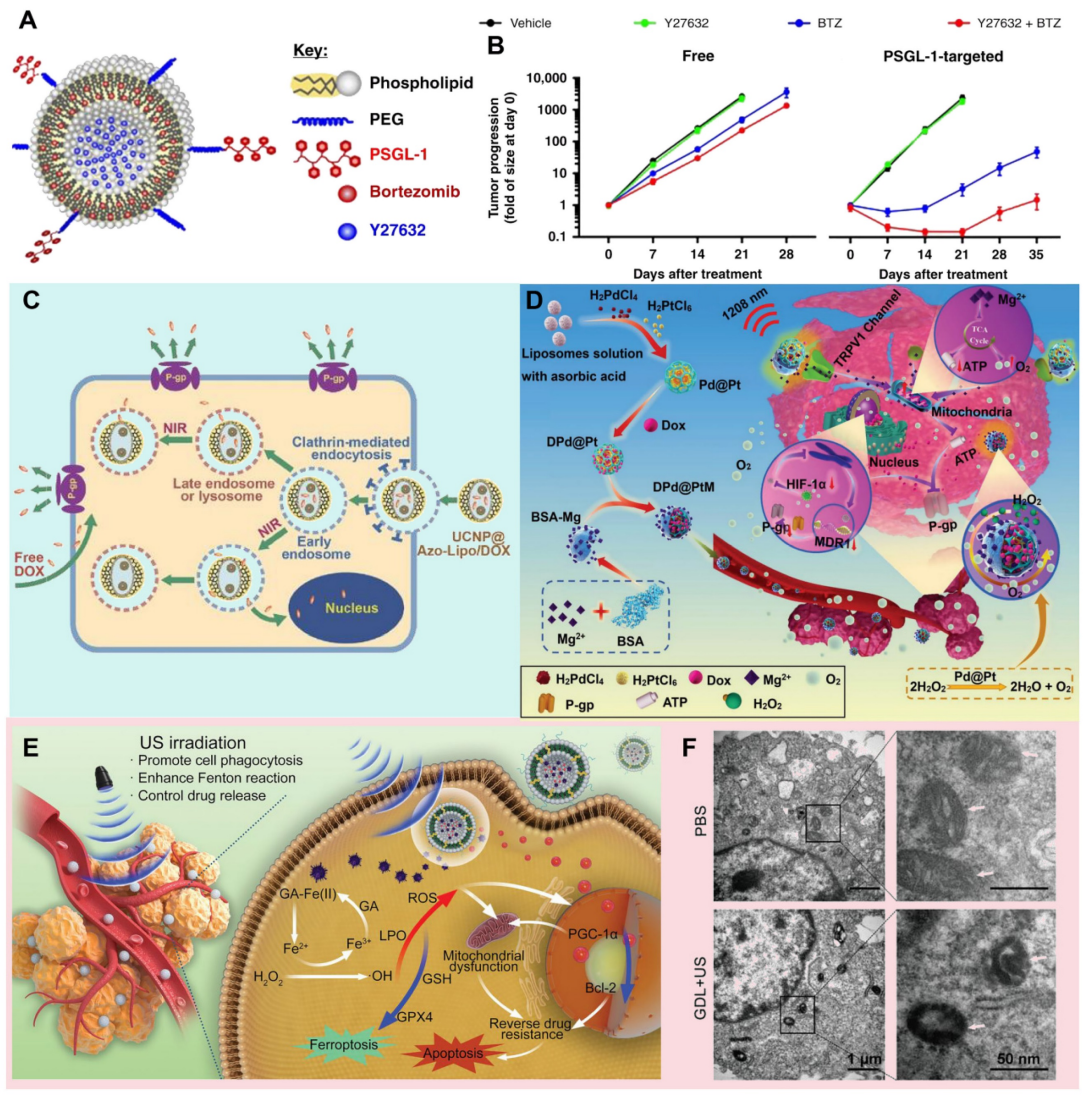
Application of lipid-based Nano-DDS against MDR tumors. **(A)** Illustration of PSGL-1 combination (BTZ and Y27632) nanoparticles. **(B)** Tumor size in mice treated with free and PSGL-1-targeted forms. Adapted with permission from 128, copyright 2020 Springer Nature. **(C)** Schematic illustration of intracellular drug delivery in the UCNP@Azo-Lipo/DOX nanosystem. Adapted with permission from 129, copyright 2016 Wiley. **(D)** Schematic illustration of the DPd@PtM nanosystem to reverse tumor MDR by delivering Mg^2+^ via photothermally activated TRPV1 ion channels to regulate the tricarboxylic acid cycle and alleviate tumor hypoxia. Adapted with permission from 130, copyright 2023 Wiley. **(E)** Schematic diagram of GA-Fe(II) nanosystem therapy: GA-Fe(II) nanosystem triggers iron death mechanism by increasing intracellular Fe(II) level, inducing lipid peroxidation, depleting GSH and inhibiting GPX4. At the same time, the synergistic effect of ultrasonic irradiation is significantly enhanced, which not only promotes the uptake of nano-liposome cells and exacerbates Fenton reaction, but also down-regulates the expression of PGC-1α and Bcl-2 through GA-Fe(II)-generated •OH, reversing drug resistance and enhancing doxorubicin-induced apoptosis. **(F)** Representative biological TEM images of cells treated with PBS and GDL+US. White arrows indicate mitochondria. Adapted with permission from 131, copyright 2022 Wiley.

Thus, the bioavailability and therapeutic effect of drugs can be ultimately improved. For example, Federico *et al.* designed a liposome nanoparticle system that encapsulates the chemotherapy drug Bortezomib (BTZ) and bone marrow microenvironment (BMME)-disrupting agents (ROCK inhibitor, Y27632) within its interior, while its outer surface is modified with the specific ligand (P-selectin glycoprotein ligand-1, PSGL-1) for targeting overexpressed P-selectin in BMME (**Figure 3A**). The direct interaction of multiple myeloma (MM) cells with stromal and endothelial cells (ECs) in BMME induces drug resistance in MM cells, which can be eliminated through the ROCK inhibitor mediated signaling cascade. In this design, PSGL-1-decorated liposomes loaded with BTZ and BMME-disrupting ROCK inhibitor Y27632 specifically target ECs, effectively block the interaction between MM cells and BMME, thereby overcoming TME-induced resistance and ultimately enhancing therapeutic efficacy (**Figure 3B**) 128. The advantage of liposomes as the drug delivery system lies not only in their flexible packaging capabilities and structural diversity, but also in their ability to be precisely designed for different therapeutic needs. Specifically, this liposome intelligent delivery system can precisely release drugs within tumor cells after the tumor site is stimulated by external stimuli (such as near-infrared (NIR) light), thus achieving specific killing of tumor cells and reducing damage to surrounding normal tissues. For example, Yao* et al.* a liposomal drug delivery system utilizing upconversion nanoparticles (UCNPs) encapsulated within azobenzene liposomal nanostructures (UCNP@Azo-Lipo), achieving the release of doxorubicin (DOX) at specific spatial locations through precise manipulation of near-infrared light. This innovative design not only effectively circumvents lysosomal capture of DOX but also mitigates the extravasation of DOX resulting from liposome decomposition and the MRP1 effect, thereby significantly enhancing drug absorption and utilization efficiency within the tumor microenvironment (**Figure 3C**) 129.

Until now, there has been an increasing number of researches on the combination of metal and liposome, which not only greatly improves the stability of the system, but also leads the revolutionary progress of drug controlled release and bioactive substance delivery technology. For example, Shao* et al.* designed a NIR light-responsive palladium-platinum liposome nanoparticle (DPd@PtM) for delivering DOX and Mg^2+^ (**Figure 3D**). DPd@PtM nanosystem reduces ATP (adenosine triphosphate) production and oxygen consumption through Mg^2+^ regulation of mitochondrial tricarboxylic acid cycle, and collaborates with its intrinsic catalase activity to inhibit the expression of Hif-1α. These mechanisms in turn inhibit the expression of P-gp protein, thus significantly enhancing the therapeutic effect of DOX 130. Similarly, Zheng* et al.* designed the US-responsive ferrous gallate (GA-Fe(II)) composite liposome nanosystem that also contained the chemotherapy drug DOX (**Figure 3E**). GA-Fe (II) nanocomplexes were uptaken by tumor cells through the synergistic effect of passive targeting and ultrasonic (US) stimulation. Subsequently, by catalyzing the overexpressed H_2_O_2_ in the TME, GA-Fe(II) nanocomplexes generate toxic •OH, which depletes GSH and raises oxidative stress (**Figure 3E**). This process leads to lipid peroxidation (LPO) and ferroptosis while limiting mitochondrial activation, ultimately making resistant cells more responsive to chemotherapeutic agents (**Figure 3F**) 131.

Liposome nanoparticles have good biocompatibility and degradability, and can be used as drug carriers to safely enter the body and target to tumor sites. By adjusting the composition and surface modification of liposomes, accurate recognition and response to TME can be achieved, thus improving the targeting and therapeutic effect of drugs. However, although liposomes can be targeted to tumor sites, the heterogeneity of tumor tissue may lead to uneven drug distribution, affecting the therapeutic effect. In addition, the metabolism and clearance mechanism of liposomes *in vivo* is not completely clear, and long-term use may have potential safety hazards.

### Polymer nanoparticles

**Figure 4:**
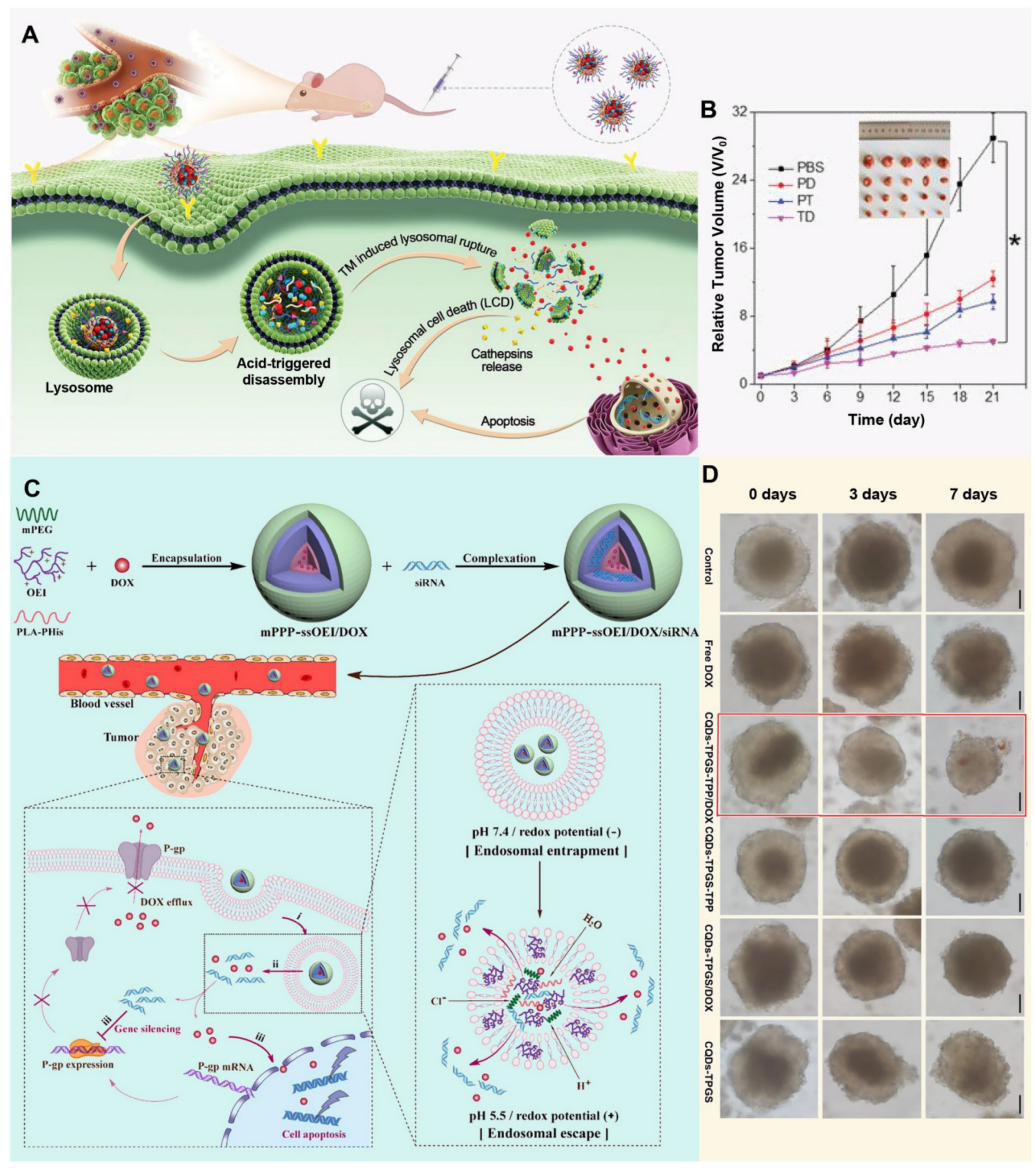
Applications of polymer nanoparticles against MDR tumors. **(A)** Schematic illustration of TD nanosystems targeting lysosomes, promoting escape of DOX from lysosomes, resulting in lysosomal cell death. **(B)** Time-dependent tumor growth curves and body weight of tumor-bearing mice under various treatments. Adapted with permission from 135, copyright 2021 Wiley. **(C)** Schematic illustration of the pH/redox dual-responsive mPPP-ssOEI/DOX/siRNA codelivery polyplex with effective endo-lysosomal escape. Adapted with permission from 136, copyright 2019 American Chemical Society. **(D)** Growth inhibition assay in MCF-7/ADR multicellular spheroids. Adapted with permission from 137, copyright 2017 American Chemical Society.

The core position of polymers in the field of pharmaceutical science is self-evident, they are not only a solid cornerstone of traditional pharmaceutical preparation, but also lead the innovation in the wave of nanomaterials technology, and directly attack the complexity and drug resistance of the TME by accurately regulating the targeting and effectiveness of drugs 132. With its excellent functionalization ability, polymer materials can skillfully construct a variety of drug carrier systems at the nanoscale, whether it is particle core encapsulation, matrix capture, chemical conjugation and surface binding. Furthermore, they can flexibly accommodate various payloads, including both hydrophobic and hydrophilic molecules, as well as cells 133. This is critical to improving the effectiveness of treatment for drug-resistant tumors. Polymer nanoparticles exhibit extraordinary drug delivery potential due to their diverse forms, such as polymers, micelles and dendrimers. They not only possess ideal properties, including biodegradability, excellent dispersion, biocompatibility, and storage stability, but also effectively protect drug activity, reduce side effects, and improve the safety and efficiency of treatment 134. Through surface modification technology, polymer nanoparticles can accurately identify and navigate to tumor cells, penetrate the barriers of the TME, and directly attack the lesion, thereby opening up a new way for the treatment of MDR tumors. When exploring new strategies for tumor treatment, accurately targeting key components of the TME has become the focus of scientists. Among them, targeted drug delivery of lysosomes in tumor cells has attracted much attention due to its unique physiological environment. For example, Li *et al.* developed pH-sensitive multifunctional nanoparticles (TD NPs) by encapsulating a lysosome-targeting, aggregation-induced-emission drug (TM) and DOX within amphiphilic polymers, specifically designed to target cancer cells and counteract drug resistance (**Figure 4A**). In the acidic lysosomal environment, DOX is released, and TM facilitates its escape from lysosomal entrapment, thereby enhancing the effective intracellular concentration of DOX. This lysosome-targeted release and escape mechanism enables DOX to reach the nucleus, effectively inhibiting the proliferation of drug-resistant MCF-7/ADR cells and reversing MDR (**Figure 4B**) 135. With a deeper understanding of cancer biology, researchers are beginning to explore more intelligent and precise micellar polymer drug delivery systems, for example, Gao *et al*. designed a nanoparticle, mPEG-b-PLA-PHis-ss-OEI, that leverages dual pH/redox responsiveness to effectively co-deliver siRNA and chemotherapeutic drugs, thereby reversing MDR in cancer cells by enhancing endolysosomal escape and facilitating the release of the therapeutic payload (**Figure 4C**) 136. The characteristics of the TME also provide new targets and strategies for cancer treatment. Zhang *et al.* designed a trackable, mitochondria-targeting drug delivery system based on self-assembled TPP-TPGS nanomicelles and fluorescent carbon quantum dots, utilizing the latter as fluorescent indicators to monitor the internalization and localization of the TPP-TPGS nanomicelles within cancer cells, specifically addressing mitochondrial targeting. By precisely targeting mitochondria and facilitating the delivery of therapeutic agents, these nanoparticles demonstrated a significant reversal of MDR in both cancer cells and their three-dimensional multicellular spheroids (**Figure 4D**) 137.

Targeting the TME with polymer nanoparticles for drug resistant tumors presents significant advantages, but also comes with some challenges 138. Its long-term biosafety and potential effects on the body need further study. At the same time, the heterogeneity of tumor tissue may cause the nanoparticles to not work effectively in some areas, and long-term use may also lead to new drug resistance problems. Therefore, when using polymer nanoparticles for tumor therapy, it is necessary to comprehensively consider its advantages and disadvantages, and constantly optimize the treatment plan to improve the therapeutic effect and reduce the potential risk.

### Inorganic nanoparticles

Inorganic nanoparticles, such as metals, metal oxides, silicon-based nanoparticles, etc., have a variety of size and composition-dependent physical properties 139. This diversity allows inorganic nanoparticles to be precisely designed for the specificity of the TME, for example, by adjusting the size, shape, and surface properties of the material to achieve targeted recognition and efficient penetration of tumor tissue 140. In addition, inorganic nanoparticles usually have a higher specific surface area and porosity, which can load more drug molecules or therapeutic agents and improve the therapeutic effect 121, 141. Inorganic nanoparticles have also shown remarkable prospects in the field of targeting TME to treat drug resistant tumors. Through surface modification and functionalization, inorganic materials can carry targeted molecules to achieve accurate identification and binding of tumor cells, thus improving the targeting and precision of therapy. To date, silicon-based nanoparticles and metal nanoparticles are the two most commonly used inorganic nanoparticles in the treatment of drug-resistant tumors.

#### Silicon-based Nano-DDS

**Figure 5:**
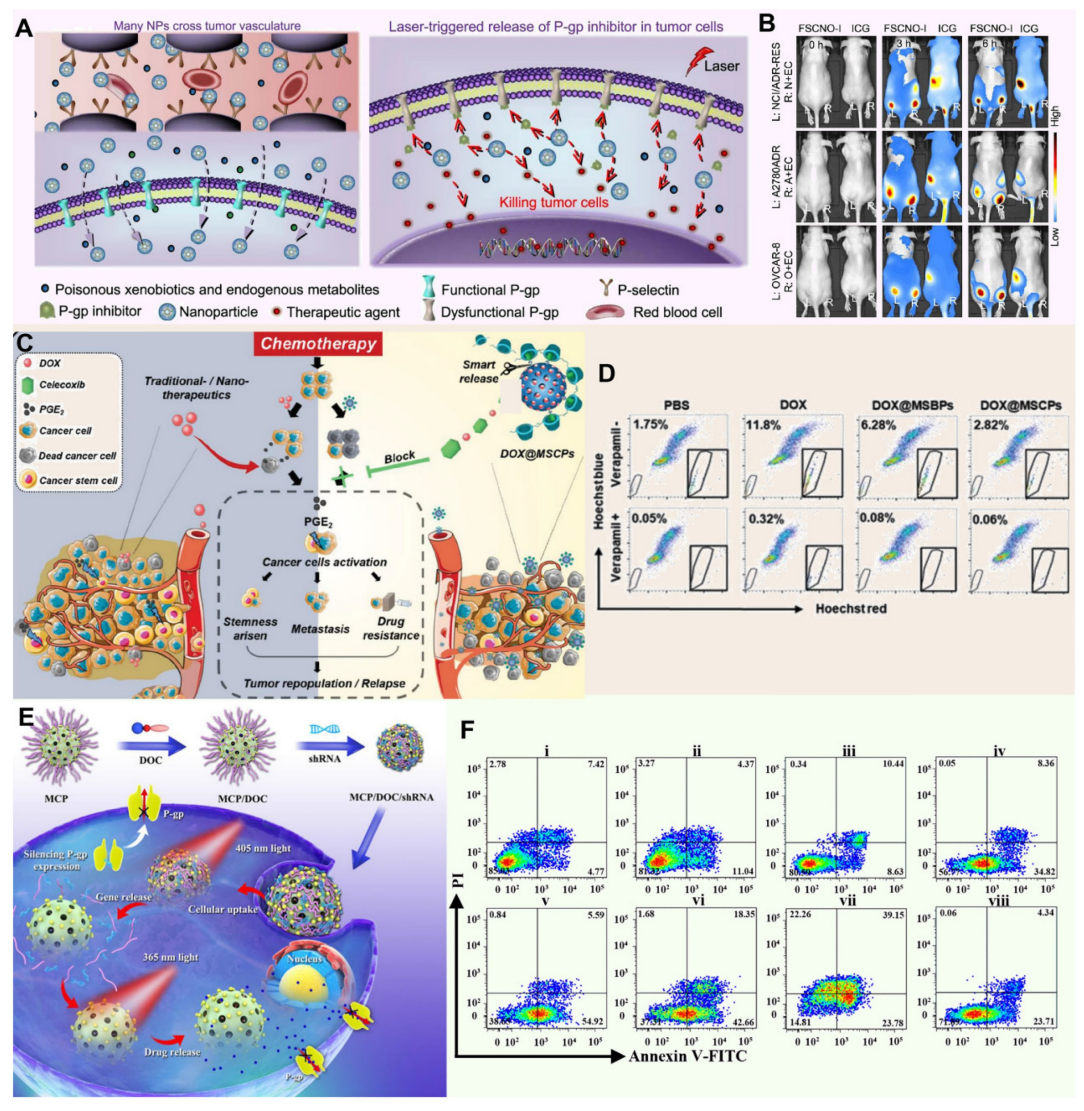
Applications of silicon-based Nano-DDS against MDR tumors.** (A)** The schematic diagram shows that FSCNO nanoparticles targeting tumor vasculature accumulate in tumor through P-selectin mediated active and EPR effect-mediated passive targeting, release P-gp inhibitors and chemotherapy drugs under NIR laser irradiation, effectively inhibit tumor P-gp function, and reduce the influence of P-gp pump on normal cells.** (B)** Imaging of mice with ICG-loaded FSCNO (FSCNO-I) nanoparticles *in vivo.* Adapted with permission from 142, copyright 2021 Springer Nature. **(C)** The schematic diagram shows that the MSCPs nanosystem blocks the COX-2/PGE2 axis, increases the sensitivity of drug-resistant cancer cells to DOX, eliminates DOX-induced cancer dryness, metastasis, and inhibits P-gp expression. **(D)** Changes in the number of cancer stem cell-like cells expressing P-gp in mice after treatment at DOX@MSCPs. Adapted with permission from 143, copyright 2019 Wiley.** (E)** Schematic illustration of sequential release of shRNA and DOX regulated by 405 and 365 nm light irradiations, using photoresponsive mesoporous silica nanoparticles as co-delivery vehicles for optimizing the synergistic therapy in multidrug-resistant cancer cells. **(F)** Flow cytometry analysis of HepG2/ADR cell apoptosis induced by different treatments, as indicated using Annexin-V-FITC/propidium iodide apoptosis detection assay. Adapted with permission from 144, copyright 2018 American Chemical Society.

Silicon-based Nano-DDS have gained widespread acceptance in biomedical research, especially in the field of drug delivery. Among these materials, mesoporous silica occupies a central position in biomedical applications. Based on the stability of silica, Wang *et al.* reported a silicon-carbon onion hybrid nanoparticle (FSCNO) modified with botryococcus polysaccharides, enabling it to specifically bind to p-selectin, which is overexpressed in tumor vasculature (Figure 5A). Under low-power near-infrared (NIR, 800 nm) laser irradiation, the FSCNO nanoparticles can precisely release P-gp inhibitors and anticancer drugs to tumor cells to overcome MDR. By minimizing the accumulation of FSCNO in normal organs and encapsulating the inhibitor within the nanoparticles, this approach maximizes protection of the P-gp function in normal organs for the exclusion of toxic exogenous metabolites, thereby reducing side effects on normal tissues (Figure 5B) 142. In the field of targeted tumor therapy, accurate identification and effective intervention of the TME is the key to improve the therapeutic effect. Based on this idea and the redox state of TME, Liu *et al.* synthesized a celecoxib-based redox-active polymer (β-cyclodextrin) to coat mesoporous silica nanoparticles (MSCP) loaded with the anticancer drug DOX (Figure 5C). As a specific COX-2 inhibitor, celecoxib blocks the COX-2/PGE2 signaling pathway, inhibiting the proliferation and metastasis of cancer stem cells. Additionally, celecoxib indirectly suppresses P-gp expression by reducing PGE2 production, thereby lowering the ability of tumor cells to expel chemotherapeutic drugs. This effect potentially reduces cancer cell regrowth while inhibiting tumor cell stemness and invasiveness (Figure 5D) 143. To address MDR in cancer, Wu* et al.* prepared photoresponsive mesoporous silica nanoparticles (PMSN) as a co-delivery carrier for P-glycoprotein short hairpin RNA and doxorubicin photocaged prodrug, enabling orthogonal and sequential release of shRNA and DOX using external light (Figure 5E). Their study revealed that the PMSN were effectively internalized by MDR cancer cells, and due to selective cleavage of coumarin and o-nitrobenzyl esters, the release of shRNA and DOX was independently regulated by 405 and 365 nm light irradiation, respectively, leading to enhanced drug retention and ultimately producing optimized and significantly improved chemotherapy effects for MDR cancer treatment both *in vitro* and *in vivo* (Figure 5F) 144.

#### Metallic nanomaterials

**Figure 6:**
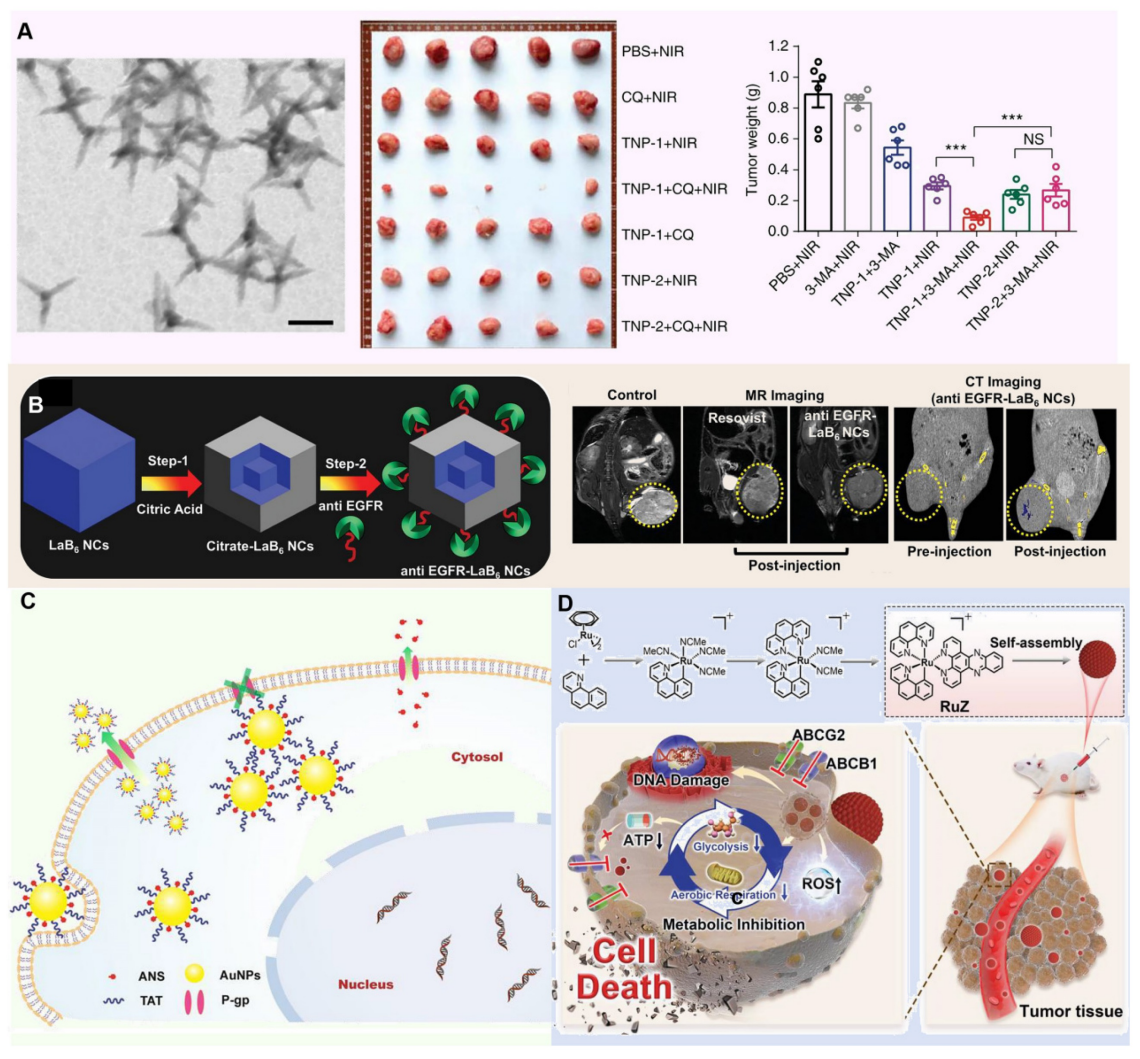
Applications of metallic nanomaterials against MDR tumors. **(A)** TEM image of CuPd TNP-1 nanoparticles and their tumor therapeutic effect. Adapted with permission from 148, copyright 2018 Springer Nature. **(B)** Schematic diagram of synthesis of anti-EGFR-LAB_6_NCs and *in vivo* T_2_-weighted MR images of mice injected with nanoparticles and MR contrast agent. Adapted with permission from 149, copyright 2020 Wiley. **(C)** Schematic diagram of ANS-TAT-AuNPs reversing MDR. Adapted with permission from 150, copyright 2017 American Chemical Society. **(D)** The schematic diagram shows that RuZ nanoparticles can effectively inhibit the growth of MDR cancer cells by inhibiting glycolysis, reducing ATP level, inducing oxidative stress and DNA damage. Adapted with permission from 151, copyright 2021 Wiley.

Metallic nanomaterials, especially iron (Fe), manganese (Mn), gold (Au), platinum (Pt) and copper (Cu), have shown great potential in the treatment of tumor MDR by their unique physicochemical properties, such as high photothermal conversion efficiency, peroxidase activity, and catalase activity, etc. 145. Until now, metallic nanomaterials have been gradually becoming a new weapon to combat this medical problem of tumor MDR. Particularly noteworthy is the photothermal effect of these materials, which under specific conditions (such as low oxygen, acidic pH and high concentrations of reducing substances), efficiently converts light energy into heat energy, either directly disrupting tumor cell structures or activating the immune response within the TME 146, 147. By precisely regulating the TME, metal nanomaterials not only enhance the therapeutic effect, but also reduce the damage to normal tissues, providing a new strategy and hope for completely conquering MDR tumors. For example, Zhang *et al.* designed copper-palladium (Cu-Pd) alloy tetrapod nanoparticles (TNP-1) with high photothermal conversion efficiency, combined with an autophagy inhibitor to combat triple-negative and drug-resistant breast cancer. Through photothermal effects, TNP-1 induces pro-survival autophagy, triggering stress responses within tumor cells. In combination with the autophagy inhibitor, these nanoparticles disrupt tumor cells' self-protective mechanisms, reduce multidrug resistance, and enhance anticancer drug efficacy, achieving substantial therapeutic effects against drug-resistant cancers (Figure 6A) 148.

Analogously, Kuthala *et al.* reported reported that anti-EGFR-modified lanthanum boride nanocubes (LaB6 NCs) exhibit a photothermal response under 980 nm and 1550 nm light irradiation, targeting EGFR. In NCI-H23 cells, these anti-EGFR-LaB6 NCs demonstrated strong absorption, effectively alleviating tumor hypoxia. Furthermore, by generating localized hyperthermia and disrupting hypoxia-driven survival pathways, the nanocubes helped reverse chemoresistance in inherently drug-resistant NCI-H23 lung cancer, supporting both diagnosis and therapy (Figure 6B) 149.

In the in-depth study of the complex mechanism of MDR, the critical inducement, tumor hypoxia environment, is specifically targeted. This harsh microenvironment not only provides shelter for the survival and proliferation of tumor cells, but also greatly weakens the therapeutic effect of traditional chemotherapy drugs, thereby becoming an important driving force for the development of MDR. In order to effectively address this challenge, many researchers used the metal's own peroxidase to design innovative nanomedicine for relieving oxygen deprivation inside tumors. For example, Wang *et al.* Prepared a drug delivery system consisting of TAT-modified gold nanoparticles (AuNPs) that efficiently deliver 2-(9-anthrylstyryl)-hydrazinocarbonamide (ANS) into cancer cells, overcoming MDR by enabling high intracellular concentrations of the anticancer agent. And the TAT-derived peptide anchored on the AuNPs facilitates membrane penetration and enhances cellular uptake, thereby reversing resistance to chemotherapy and restoring the effectiveness of treatment in MDR cancer (**Figure 6C**) 150. Furthermore, Ja *et al.* developed a self-assembled ring-shaped RuZ metallic complex with an octahedral structure that minimizes interaction with ABCB1 and ABCG2 transporters, allowing for increased retention in drug-resistant cancer cells. The high electron density at the metal center reduced oxygen consumption, while the complex's redox activity inhibited glycolysis and lowered ATP levels, effectively targeting MDR mechanisms in cancer cells (**Figure 6D**) 151.

### Hydrogels

**Figure 7:**
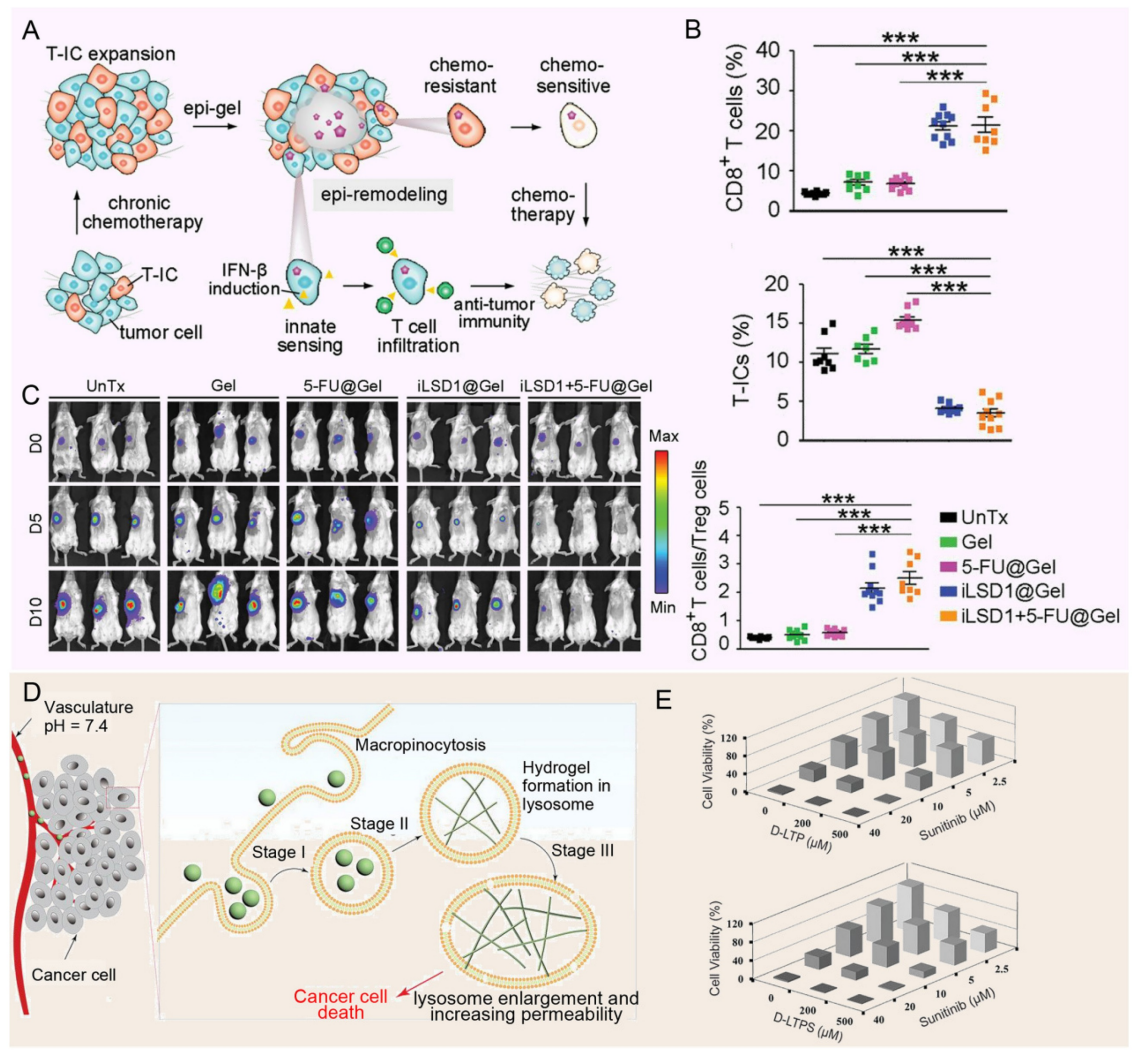
Applications of hydrogels against MDR tumors. **(A)** The schematic depicts the consequences of Epi-gel on chemo-resistant TNBC. By locally and continuously inhibiting LSD1, Epi-gel diminishes T-IC prevalence, enhances chemotherapeutic responsiveness, and triggers innate immune activation.** (B)** Live images of mice after different treatments. **(C)** The proportion of tumor-initiating cells in drug-resistant breast cancer treated with no-load hydrogel, the infiltration of CD8^+^ T cells in drug-resistant breast cancer after treatment. Adapted with permission from 156, copyright 2021 Wiley. **(D)** Schematic diagram shows upon endocytosis by cancer cells (Stage I), LTP oligomers accumulate within lysosomes, undergoing proton-induced phase transformation into a nanofibrous hydrogel (Stage II). This transformation subsequently prompts lysosome enlargement (Stage III), triggering LMP and ultimately leading to cancer cell death. **(E)** Combination therapy of peptides D-LTP (or D-LTPS) and sunitinib against SK-OV-3 cells after 48 h incubation. Adapted with permission from 157, copyright 2021 Wiley.

As a cutting-edge drug carrier for the treatment of solid tumors, hydrogels have been gradually showing their unique advantages and potential 152, 153. Its distinctive gel structure not only offers a stable platform for controlled drug release but also significantly enhances the bioavailability of drugs within tumor lesions through localized administration. This approach effectively minimizes the widespread distribution of drugs throughout the body, thereby drastically reducing non-specific damage and mitigating side effects to normal tissues 154, 155. Ma *et al.* designed a hydrogel platform that targets lysine-specific demethylase 1 (LSD1) within the TME, providing localized, continuous, and controlled delivery of therapeutic agents (**Figure 7A**). By detecting elevated ROS in the TME, the platform releases the LSD1 inhibitor (GSK-LSD1), which remodels the epigenome to reduce CSC stemness. This approach also activates tumor cell immunity, effectively reversing MDR. A single dose of the hydrogel patch containing GSK-LSD1 and 5-FU successfully inhibited tumor growth, postoperative recurrence, and metastasis (**Figure 7B and 7C**) 156. At the same time, the study by Wang *et al.* introduced a novel approach for using hydrogels in the treatment of MDR tumors. They designed pH-responsive hexapeptide (LTP)-based nanofiber hydrogels that undergo proton-induced phase transition in the acidic lysosomal environment, leading to lysosome enlargement in cancer cells (**Figure 7D**). This enlargement enhances the accumulation of chemotherapy drugs, improving their effectiveness and overcoming multidrug resistance (MDR), thereby increasing the ability of the drugs to kill MDR tumor cells (**Figure 7E**) 157. The ability of hydrogels to provide continuous and controlled drug release is particularly important for diseases requiring long-term treatment or where drug concentrations need to be maintained. Its unique gel structure is able to form a reservoir at the tumor site and gradually release the drug, thereby extending the duration of drug action and reducing the frequency of administration 154, 158. In addition, local administration of hydrogels can significantly improve the bioavailability of drugs in tumor lesions, while reducing the distribution of drugs throughout the body and the toxic side effects on normal tissues 155. However, the mechanical properties of hydrogels are relatively weak and may not withstand the mechanical challenges of complex environments in the body, such as blood flow impact or tissue movement. This leads to a decrease in the stability of the hydrogel in the body, affecting the sustained release effect of the drug. In addition, the distribution and metabolism of hydrogels in the body may be affected by many factors, such as blood flow, tissue permeability, etc. These factors may lead to the uneven distribution of hydrogels in the body, affecting the targeting and therapeutic effect of drugs. To sum up, hydrogel as a drug carrier for the treatment of solid tumors has many advantages, but there are also some shortcomings that need to be overcome. Future studies should focus on optimizing the properties and preparation processes of hydrogels to improve their efficacy and safety in tumor therapy.

### Biomimetic nanoparticles

**Figure 8:**
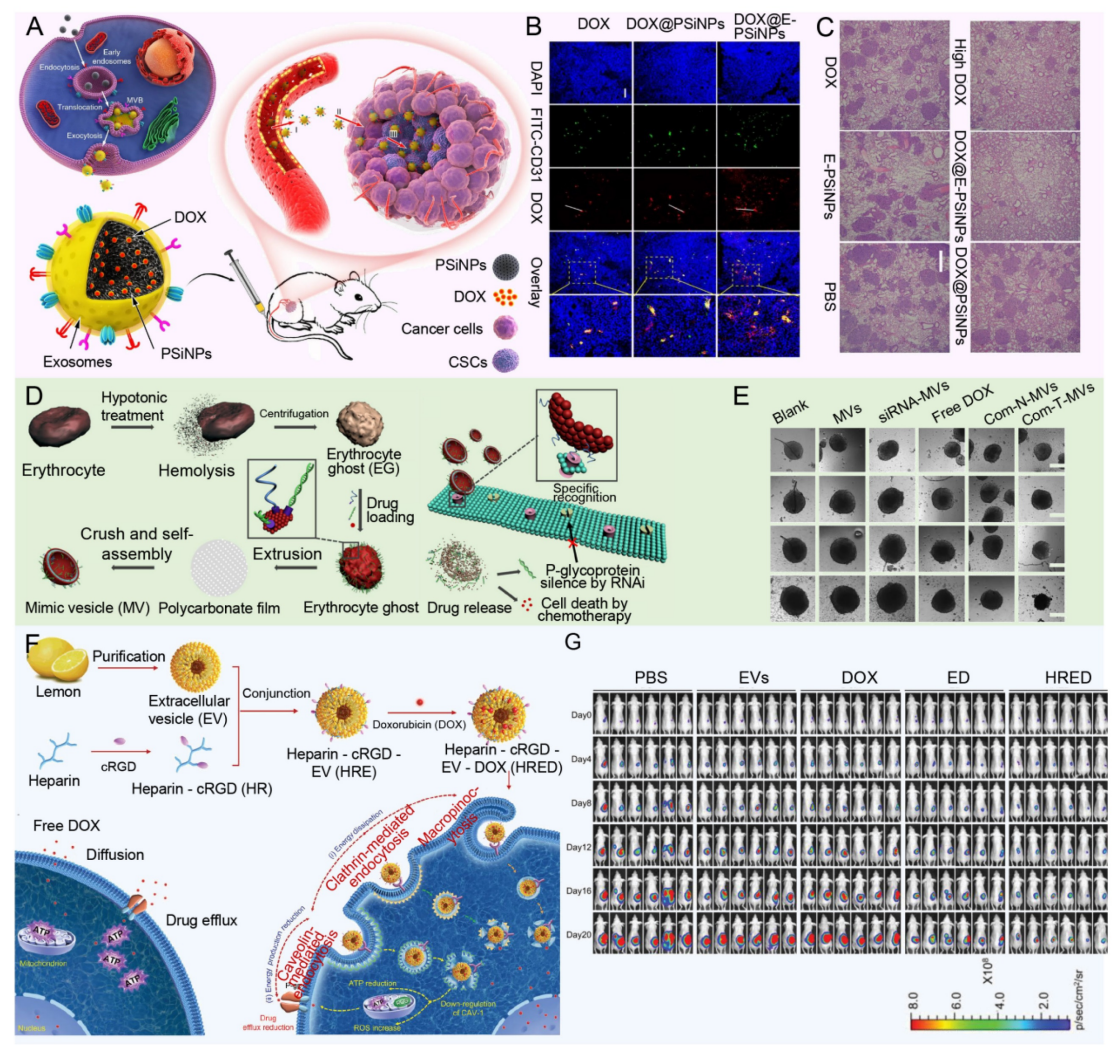
Applications of biomimetic nanoparticles against MDR tumors. **(A)** The diagram illustrates the process of DOX@E-PSiNP formation after DOX@PSiNPs is endocytosed into cancer cells after incubation. **(B)** Distribution of DOX in tumor of mice after injection of different nanoparticles. **(C)** H&E staining of the lungs of mice after different treatment. Adapted with permission from 162, copyright 2019 Springer Nature. **(D)** Schematic illustration of siRNA/DOX co-loaded MV overcoming drug resistance and synergistically killing MDR tumors through P-gp silencing and DOX-induced growth inhibition. **(E)** Representative optical images of multicellular spheres treated with different nanosystems. Adapted with permission from 163, copyright 2019 American Chemical Society. **(F)** The diagram shows that lemon-derived HRED nanotherapeutic drugs (Heparin-CrGD modified EV-loaded DOX) enhance the uptake of cancer cells through diversified endocytic pathways, down-regulate CAV-1 to reduce ATP production and increase ROS levels, effectively expend energy and inhibit drug efflux, thus overcoming MDR in cancer. **(G)** Bioluminescence imaging *in vivo* imaging system of each group of nude mice with ovarian cancer during treatment. Adapted with permission from 164, copyright 2022 Wiley.

Bionic nanoparticles build an innovative and efficient drug delivery platform by cleverly blending the unique functions of natural biomaterials, such as the biocompatibility, circulatory stability and targeting of cell membranes of red blood cells, cancer cells, platelets or white blood cells, with the engineered versatility of synthetic nanoparticles 159-161. This design not only enhances the stability and compatibility of the nanoparticles in the organism, but also significantly improves their ability to target tumor tissue, thereby reducing the side effects of the drug on non-targeted tissues. For example, Yong *et al.* developed exosome biomimetic nanoparticles loaded with DOX and luminescent porous silicon nanoparticles (DOX@E-PSiNPs) (**Figure 8A**). The nanoparticle system reverses drug resistance by enhancing targeted drug delivery and increasing cytotoxic effects specifically within the TME, where it accumulates at high concentrations and penetrates deeply into tumor tissues, bypassing barriers that often limit conventional chemotherapy effectiveness (**Figure 8B**). By preferentially targeting CSCs, which are key drivers of tumor recurrence, metastasis, and resistance, DOX@E-PSiNPs effectively disrupts the cellular mechanisms, such as MDR efflux pumps, that cancer cells use to evade chemotherapy, thereby reducing the potential for relapse and improving treatment outcomes (**Figure 8C**) 162. In addition, the modified aptamers on the surface of nanoparticle further improve the targeting and efficacy of drug delivery. In another interesting study, Wang *et al.* developed a multifunctional siRNA-N-MV delivery system by modifying targeted molecules, such as AS1411 aptamers, onto erythrocyte membrane-simulated vesicles, allowing the system to specifically recognize and target tumor cells with high nucleolin expression (**Figure 8D**). This system efficiently delivered both P-glycoprotein siRNA and DOX to MDR tumor cells, reversing drug resistance by silencing P-gp expression, thereby overcoming a key mechanism that tumor cells use to evade chemotherapy (**Figure 8E**) 163.

Although cancer cell derived vesicles show strong homing ability in drug delivery, their preparation process is complex and the yield is limited, which limits their application to some extent. In contrast, plant-derived vesicles, such as grapefruit, lemon and ginger, are emerging as excellent candidates for the delivery of various therapeutic agents due to their advantages of mass production and simple encapsulation procedures. For example, Xiao *et al.* designed a lemon-derived extracellular vesicle (EV) drug delivery system (HRE-DOX) designed to overcome cancer multidrug resistance (MDR) by dissipating intracellular energy through enhanced endocytosis (**Figure 8F**). By incorporating heparin-cRGD (HR) onto the surface of the lemon-derived EVs to deliver doxorubicin (DOX), the HRED system gains anti-complement activation properties and targeting capabilities, enabling it to enter DOX-resistant cancer cells via multiple pathways, including caveolin-mediated endocytosis, clathrin-mediated endocytosis, and macropinocytosis, thereby substantially depleting cellular energy. Furthermore, during caveolin-mediated endocytosis, HRED downregulates caveolin-1 (CAV-1) expression, reducing ATP production while increasing ROS levels, which, combined with energy dissipation and ATP reduction, enables this system to effectively reverse cancer MDR (**Figure 8G**) 164. As a cutting-edge technology in drug delivery systems, biomimetic nanoparticles have the advantage of skillfully integrating the dual characteristics of natural biomaterials and synthetic nanoparticles. On the one hand, they inherit the biocompatibility, circulatory stability, and natural targeting abilities of natural biomaterials, such as cell membranes, thereby rendering the nanoparticles more efficacious *in vivo*. This enables them to evade clearance by the immune system, prolong their circulation time, and precisely target tumor tissues 159. On the other hand, the introduction of synthetic nanoparticles gives them a powerful loading capacity, controlled release properties, and a modifiable surface, allowing drugs to be efficiently encapsulated inside the nanoparticles and released at a specific time and place according to therapeutic needs, thus enabling precise treatment of diseases 160. However, bionic nanoparticles also face some challenges and drawbacks. Firstly, although its design is inspired by nature, it is still a huge challenge to accurately simulate and optimize these natural processes in a laboratory environment. This involves many aspects such as complex biochemical reactions, precise control of nanostructures, and maintenance of biological activity. Secondly, the preparation process of biomimetic nanoparticles is usually complicated and costly, which limits their promotion in mass production and clinical applications to a certain extent. In addition, despite their good biocompatibility, they still have the potential to trigger immune reactions or other unforeseen biosafety issues when used in long-term or high doses. Therefore, in promoting the further research and application of biomimetic nanoparticles, it is necessary to comprehensively consider their advantages and disadvantages, continuously optimize the preparation process, strengthen the biosafety assessment, and explore more potential applications in the treatment of diseases.

### DNA nanocarriers

**Figure 9:**
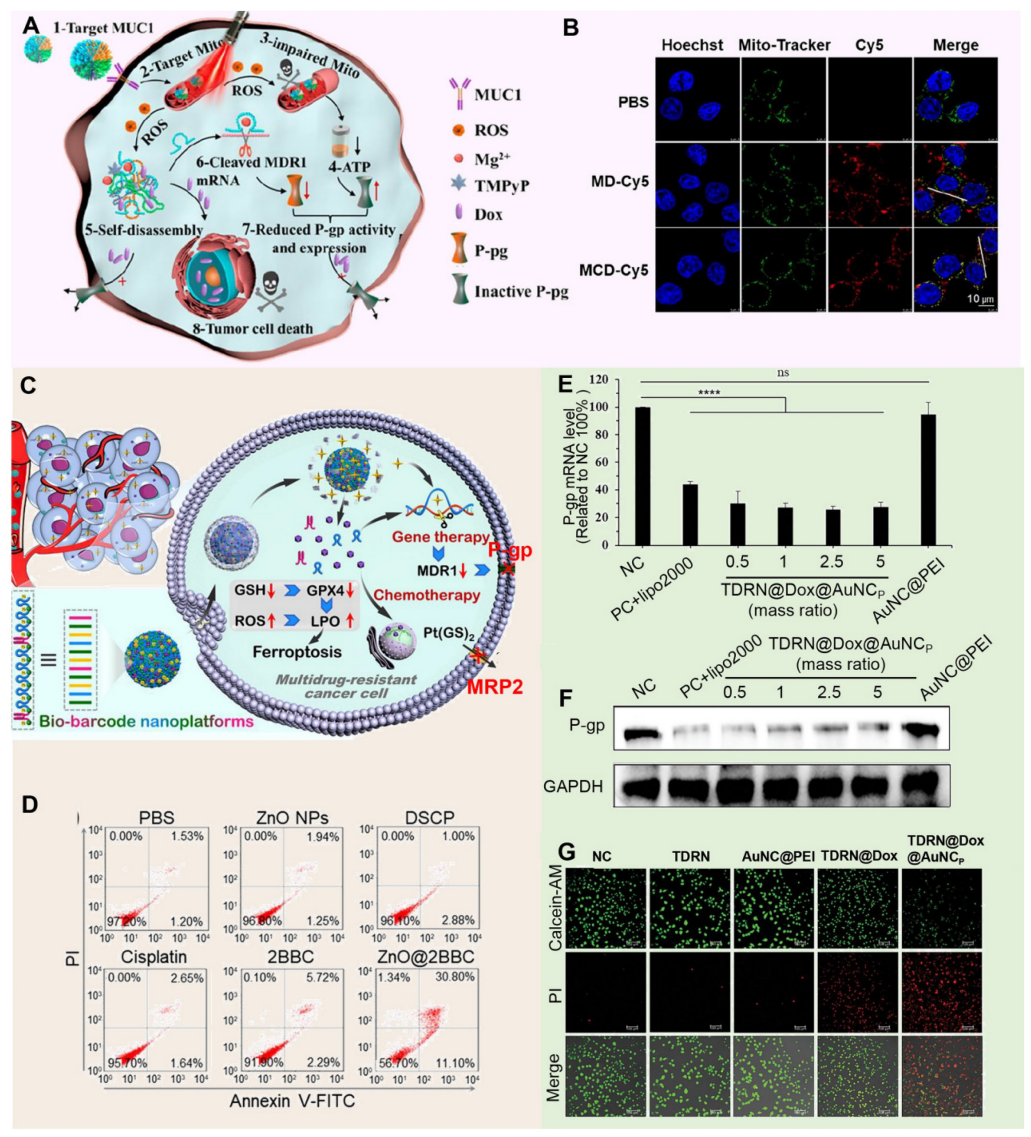
Applications of DNA nanocarriers against MDR tumors. **(A)** Schematic diagram shows that MCD@TMPyP4@DOX reverses breast cancer drug resistance through a dual pathway of gene regulation and mitochondrial damage, targeting MCF-7/ADR cell mitochondria, triggering ROS release by near-infrared light to destroy mitochondria and reduce ATP production to inhibit P-gp activity, while releasing DNAzyme and DOX. DNAzyme silenced MDR1 mRNA and inhibited P-gp expression, jointly prevented DOX outflow and enhanced anti-tumor effect. **(B)** Colocalization of different treated nanoparticles in MCF-7/ADR cells. Adapted with permission from 167, copyright 2023 American Chemical Society.** (C)** The schematic diagram shows that the FNA engineered nanoplatform achieves synergistic enhancement of MDR cancer by down-regulating the expression of Egr-1 and MDR1, inhibiting P-gp, and reshaping the tumor internal environment to induce iron death. **(D)** Apoptosis of cells treated with different nanosystems was analyzed by flow cytometry using Annexin V-FITC/PI double staining. Adapted with permission from 168, copyright 2023 American Chemical Society. **(E)** Real-time qPCR analysis of P-gp mRNA silencing efficiency after treatment with TDRN@Dox@AuNCpNA nanosystem. **(F)** Western blot analysis of P-gp protein expression after TDRN@Dox@AuNCp treatment. **(G)** Imaging of living and dead cells to validate the cytotoxicity of different nanosystems. Adapted with permission from 169, copyright 2024 Wiley.

DNA nanostructures with controllable size and shape show broad application prospects in the field of drug delivery due to their unparalleled programmability, excellent biocompatibility, and high biophysical regulatory ability 165, 166. These nanostructures can not only be precisely designed to fit complex therapeutic needs, but can also be stable in living organisms and perform their functions efficiently. In the year of 2023, Wang *et al.* developed a multifunctional DNA nanomedicine, MCD@TMPyP4@DOX, specifically designed to overcome MDR by targeting mitochondria (**Figure 9A**). This system uses an MUC1 aptamer for cell membrane targeting and a CytC aptamer for mitochondrial localization, and, upon laser irradiation, generates ROS to destroy DNA and release both DOX and P-gp DNAzymes (**Figure 9B**). Additionally, MCD@TMPyP4@DOX self-decomposes in the acidic tumor environment, releasing Mg^2+^-assisted DNAzymes that silence MDR1 mRNA and downregulate P-gp, while mitochondrial-targeted photodynamic therapy disrupts mitochondrial function, depleting ATP and further inhibiting P-gp activity, thus effectively reversing drug resistance 167. Meanwhile, Zhi *et al.* designed a ZnO@BBCs nanoplatform that combines cisplatin prodrugs, AS1411 aptamers targeting nucleolin-overexpressing A549/DDP cells, and Egr-1 mRNA-cleaving DNAzymes to effectively counteract MDR (**Figure 9C**). Upon accumulation at the tumor site, the ZnO@BBCs release Zn^2+^ ions in response to the acidic microenvironment, facilitating the efficient cytoplasmic release of DNA nanostructures and triggering localized chemotherapy activation. The liberated DNAzymes subsequently downregulate Egr-1 and MDR1 mRNA, effectively inhibiting key resistance pathways and sensitizing tumor cells to chemotherapeutic agents (**Figure 9D**) 168. It should be noted that although the traditional DNA or RNA nanostructure is easy to prepare, it has the limitation of static and lack of dynamic response, which easily leads to the degradation of cargo oligonucleotides by nucleases *in vivo*. Therefore, researchers began to pay attention to the stable structure of the DNA or RNA scaffold itself and the use of its natural cavity. For example, Chen *et al.* constructed stimulus-responsive tetrahedral DNA-RNA nanocages (TDRN@DOX@AuNCp) specifically designed to reverse MDR in tumor cells by co-delivering P-gp siRNA and DOX (**Figure 9E**). These nanocages utilize cleavable disulfide bonds to protect siRNA from degradation, ensuring stable delivery to MDR cells, where they effectively downregulate P-gp expression (**Figure 9F**). By silencing P-gp and thereby reducing the cells' drug efflux capacity, TDRN@DOX@AuNCp significantly enhances the intracellular retention of DOX, overcoming MDR mechanisms and resulting in potent anti-tumor effects (**Figure 9G**) 169. As an innovative drug delivery system, DNA nanocarriers take advantage of DNA's programmability, biocompatibility, and stability to design complex nanostructures tailored to therapeutic requirements. They possess the capability to encapsulate protective drugs, precisely target tumor cells, and exhibit responsive release properties. It can encapsulate protective drugs, accurately target tumor cells, and has responsive release properties 170. However, the preparation process is complex, the cost is high. Meanwhile, due to the behavior of organisms *in vivo* is affected by multiple factors, the long-term or high dose use may cause biosafety issues. Therefore, it is necessary to optimize the design and preparation process of DNA nanocarriers, as well as to strengthen their evaluation to guarantee their safe and effective application in clinical practice.

## Clinical trials of Nano-DDS in targeting cancer MDR

In recent years, Nano-DDS have made significant clinical progress in overcoming tumor MDR. Up to now, a variety of nanomaterials have been successfully applied in clinical settings. For example, Doxil® (pegylated liposome doxorubicin) is the first nanomedicine approved by the U.S. Food and Drug Administration (FDA) for the treatment of multiple malignancies, including drug-resistant ovarian cancer and metastatic breast cancer. In a Phase III randomized trial involving patients with platinum-resistant ovarian cancer, Doxil® demonstrated a longer median progression-free survival (PFS) of 22.0 weeks, compared to 16.0 weeks for topotecan. The overall response rate (ORR) in the Doxil® group was 19.7%, comparable to 17.0% in the topotecan group. However, Doxil® exhibited a better safety profile with lower hematological toxicity. Through liposomal encapsulation, Doxil® utilizes the EPR effect to increase drug accumulation in tumor tissues, reduce systemic toxicity, and help overcome P-gp-mediated drug efflux mechanisms 171, 172.

As another exciting example, Abraxane® (albumin-bound paclitaxel nanoparticles) is approved for the treatment of metastatic breast cancer, non-small cell lung cancer (NSCLC), and pancreatic cancer. In a Phase III study, Abraxane® showed a higher ORR (33% vs. 19%) in patients with metastatic breast cancer compared to solvent-based paclitaxel, as well as a significantly longer median time to tumor progression (23.0 weeks vs. 16.9 weeks). By binding paclitaxel to albumin nanoparticles, Abraxane® takes advantage of the natural transport pathway of albumin, enhancing drug delivery efficiency at the tumor site, avoiding the allergic reactions and toxicity associated with traditional solvents, and helping to bypass drug expulsion mechanisms of drug-resistant cancer cells 173. In addition, as a nanometer-scale macromolecular micellar paclitaxel, Genexol-PM® is approved in South Korea for the treatment of metastatic breast cancer and NSCLC. In a Phase II trial, Genexol-PM® showed an impressive ORR of 58%, a median PFS of 8.0 months, and low toxicity 174. The formulation utilizes polymer micelles to improve the water solubility and stability of paclitaxel, enhancing drug concentration in tumor tissues and reducing the occurrence of drug resistance.

As another nanodrug approved by the FDA, Onivyde® (Irinotecan Liposome injection) is used in combination with fluorouracil and leucovorin for the treatment of metastatic pancreatic cancer. In the NAPOLI-1 Phase III clinical trial, the Onivyde® combination therapy significantly improved patients' overall survival (OS) (6.1 months vs. 4.2 months) and PFS (3.1 months vs. 1.5 months) 175. Moreover, BIND-014, which consists of PSMA-targeting docetaxelamine granules, is a docetaxel therapy specifically designed to target prostate-specific membrane antigen (PSMA). This targeting mechanism enhances the delivery of the drug to tumor cells. In a Phase I clinical trial, BIND-014 demonstrated antitumor activity in patients with advanced solid tumors, with partial responses in some, including those with metastatic castration-resistant prostate cancer. By loading docetaxel into PSMA-targeted nanoparticles, BIND-014 increased drug concentration within resistant tumor cells, showing promise in overcoming MDR 176. In short, Nano-DDS have already demonstrated numerous successful clinical trials, and they hold the potential to serve in overcoming tumor drug resistance in the future.

## Challenges and opportunities

The progress of nanoparticle-based drug delivery in tumor therapy is reflected not only in technological breakthroughs but also in the deeper understanding of tumor biology. Now, scientists are exploring the complex interactions between the TME and Nano-DDS, aiming to design smarter and more efficient nanomedicines. For example, by mimicking the surface properties of tumor cells, Nano-DDS can evade immune system clearance more effectively, enabling longer circulation times and more precise tumor targeting 177. In materials science, the development of new Nano-DDS offers more possibilities for tumor treatment. Nanomaterials with unique optical, magnetic, or thermal properties can be used for tumor imaging and diagnosis, and also trigger drug release through physical stimuli (e.g., light, magnetic fields, heat), enabling more precise therapeutic interventions 178-180. Moreover, surface modification and functionalization of nanomaterials allow them to carry various therapeutic molecules, such as chemotherapy drugs, immunomodulators, and gene therapy carriers, enabling multi-target, multi-mechanism combined therapies to overcome tumor MDR 181.

However, the widespread application of Nano-DDS in tumor therapy still faces challenges. Beyond biosafety and controlled drug release, large-scale production, quality control, and cost-effectiveness remain key barriers to clinical adoption. Additionally, the long-term effects and potential risks of nanomaterials *in vivo*, such as organ accumulation and changes in biological distribution, require further investigation. Nonetheless, with increasing interdisciplinary collaboration and technological innovation, the future of Nano-DDS in targeting tumor microenvironments for treating multi-drug-resistant tumors looks promising. We expect to see more innovative nanotechnology-based therapies enter clinical trials and applications, offering personalized, precise, and effective treatment options for cancer patients. At the same time, research into nanoparticles in cancer therapy will deepen our understanding of tumor biology, drug delivery systems, and nano-biological interactions, potentially leading to revolutionary changes in cancer treatment.

## Conclusion

In the field of cancer therapy, MDR remains one of the main factors limiting therapeutic efficacy, often leading to treatment failure and relapse. This significant challenge necessitates innovative approaches to overcome resistance mechanisms in cancer cells. Nano-DDS targeting the TME offer a promising solution to this problem, showing great potential and broad application prospects.

These Nano-DDS, including liposomal nanomaterials, polymer nanomaterials, inorganic nanomaterials such as silicon-based and metallic materials, hydrogels, biomimetic nanomaterials, and DNA nanomaterials, possess unique physical, chemical, and biological properties. Their nanoscale size and surface functionalities enable them to interact intimately with the TME, allowing for precise targeting and enhanced penetration into tumor tissues. By accurately acting on the TME, Nano-DDS can achieve more effective treatment of MDR tumors. By employing diverse mechanisms such as targeted drug delivery, photothermal therapy, catalytic reactions, and immune regulation, these Nano-DDS constitute a multifaceted treatment system. For instance, targeted drug delivery enhances the concentration of chemotherapeutic agents at the tumor site while minimizing side effects on healthy tissues. Photothermal therapy utilizes nanomaterials to convert light energy into heat, selectively destroying cancer cells. Catalytic reactions can induce the generation of reactive oxygen species within the tumor, leading to cancer cell apoptosis. Immune regulation involves modulating the immune system to recognize and attack tumor cells more effectively. Collectively, these strategies provide new methods and avenues for cancer treatment. However, while Nano-DDS targeting the TME hold significant potential for treating MDR tumors, they also face challenges that need to be addressed and improved upon.

In summary, with the continuous advancement of nanotechnology and a deeper understanding of the TME, it is believed that nanomaterials will play an increasingly important role in tumor treatment. This progress brings new hope for overcoming MDR in cancer therapy, potentially improving outcomes for more patients in the future.

## Figures and Tables

**Table 1:** Nano-DDS are used to combat MDR tumors

Nano-DDS	Nanostructure	Delivered drug	Antitumor effect	Tumor model	Ref.
Lipid-based Nanosystem	Liposomes	Bortezomib + ROCK inhibitor + P-selectin glycoprotein ligand-1	Regulates cytokines and chemokines present in the bone marrow environment	MM.1S-GFP- Luc (Myeloma)	128
	Upconversion nanoparticles + Azobenzene liposomes	Doxorubicin	Aiding drug lysosomal escape	MCF-7/ADR (Breast cancer)	129
	Magnesium ion + Pd@Pt liposome-nanostructure	Doxorubicin	Alleviates tumor hypoxia, blocks ATP production	MDA-MB- 231 (Breast cancer)	130
	Gallic acid-ferrous + liposomes	Doxorubicin	Induce iron death, Catalytic tumor of H_2_O_2_	MCF-7/ADR (Breast cancer)	131
Polymer nanoparticles	Lysosome targeting drug + amphiphilic polymer	Doxorubicin	Release drugs in response to the acidic lysosomal environment	MCF-7/ADR (Breast cancer)	135
	Triphenylphosphine - Glucan - sodium thiolate (TPP-DEX-TK) conjugates + Hyaluronic acid	Doxorubicin	Release drugs in response to ROS, induces mitochondrial dysfunction	MCF-7/ADR (Breast cancer)	136
	mPEG-PLys-AA + biodegradable amphiphilic polymer	Iron death inducer (RSL3) + Doxorubicin	Triggers drug release in response to intracellular GSH and enhances iron death potency	NCI-ADR/Res (ovarian cancer)	137
	β-lapachone + poly(ethylene glycol)- poly[2-(methylacryloyl)ethylnicotinate] polymer	prodrug BDOX + β-lapachone	Adjust NAD(P)H:quinone oxidoreductase-1 to generate ROS	MCF-7/ADR (Breast cancer)	138
Silicon-based nanosystem	Carbon nano-onion + silica + fucoidan	HM30181A + Doxorubicin	Precise control of P-gp inhibitors and chemotherapy drugs	NCI/ADR-RES , A2780ADR, OVCAR-8 (Human ovarian adenocarcinoma)	142
	Silica nanoparticles + poly(β-cyclodextrin)	Doxorubicin + Celecoxib	Decreased cancer stemness, metastasis , decreased P-gp expression	4T1 (Breast cancer)	143
	Chitosan + Pt nanoparticles + zinc-doped mesoporous silica nanocarriers	Doxorubicin	Release the drug under acidic conditions	MCF-7/ADR (Breast cancer)	144
Metallic nanomaterial	Copper-palladium alloy tetrapod nanoparticles	-	Promote autophagy of tumor cells	4T1, MCF-7/ADR (Breast cancer)	148
	Unprecedented lanthanum hexaboride nanocubes + anti epidermal growth factor receptor	-	Overcoming tumor hypoxia	NCI-H23 (Lung cancer)	149
	Branched gold nanoshells + catalase	Indocyanine green + Paclitaxel	Enhanced photodynamic therapy, Alleviate tumor hypoxic environment	U14 (Cervical cancer)	150
	Cyclometalated Ru(II) complexes	-	Reduces oxygen consumption and inhibits glycolysis	MDA-MB-231/ADR (Breast cancer)	151
Hydrogel	Hydrogel	5-fluorouracil + Lysine-specific demethylase 1 inhibitor (GSK-LSD1)	Sensing high levels of ROS release drugs in TME	4T1/ADR (Breast cancer)	156
	PH-responsive hexapeptide	-	Use of lysosomal acidification to promote cell death	SK-OV-3 (Ovarian cancer), HeLa (Cervical cancer)	157
	Methacrylate gelatin + magnetic nanoparticles	P-gp antibody	Capture resistance cell	K562/ADM (Leukemia)	158
Biomimetic nanoparticle	Luminescent porous silicon nanoparticles + tumor cell-exocytosed exosome-sheathed	Doxorubicin	Killing CSCs	H22 (Liver cancer)	162
	Erythrocyte-derived mimic vesicles + P-gp siRNA + AS1411 aptamer	Doxorubicin	Targeted delivery drug	MCF-7/ADR (Breast cancer)	163
	Llemon-derived extracellular vesicles + heparin-cRGD	Doxorubicin	Dissipate intracellular energy, dissipate intracellular energy	SKOV3/DOX (Ovarian adenocarcinoma)	164
DNA nanocarriers	mucin 1 aptamer + cytochrome C aptamer + TMPyP4	Doxorubicin	Damaged mitochondrial gene	MCF-7/ADR (Breast cancer)	167
	Functional nucleic acids + AS1411 aptamers + ZnO nanoparticles	Platinum(IV) prodrug	Increase oxidative stress and induce iron death	A549/DDP (Lung adenocarcinoma)	168
	DNA-RNA nanocages + gold nanocluster	P-gp siRNA + Doxorubicin	Induced apoptosis	HeLa/ADR (Cervical cancer)	169
